# The COVID-19 pandemic’s effects on mental and psychosocial health in the Philippines: A scoping review

**DOI:** 10.1017/gmh.2024.14

**Published:** 2024-02-08

**Authors:** Joanne Michelle F. Ocampo, Raisa Alexis N. Santos, Jesus Emmanuel A.D. Sevilleja, Christian T. Gloria

**Affiliations:** 1 Department of Population and Family Health, Columbia University Mailman School of Public Health, New York, NY, USA; 2 Department of Sociomedical Sciences, Columbia University Mailman School of Public Health, New York, NY, USA; 3 National Center for Mental Health, Mandaluyong, Philippines

**Keywords:** COVID-19, mental health, psychosocial health, LMICs, Philippines

## Abstract

Low- and middle-income countries (LMICs) remained at elevated risk for the effects of the COVID-19 pandemic because of persistent stressors to their health systems. Simultaneously facing high infection rates, strict containment measures and natural disasters, the Philippines provides important grounds for health research in LMICs. This review examined how the COVID-19 pandemic affected mental and psychosocial health in the Philippines. This scoping review included literature in English from 2020 to mid-2022 from PubMed, PsycInfo and SCOPUS, and used the PRISMA-ScR and PCC-question model. Two independent reviewers conducted blind article screening and data extraction using COVIDENCE software, followed by consensus building, data charting and analyses. This work identified 405 publications across PubMed (*N* = 56), PsycInfo (*N* = 106) and SCOPUS (*N* = 243), of which 76 articles addressed the Philippines. Article types included 54 research articles, 10 opinion pieces, 4 literature reviews, 6 letters to journals, 1 study protocol and 1 other report. These findings focused primarily on health professionals (*N* = 23) and educators/learners (*N* = 22) and reported mostly on moderate-to-severe clinical outcomes such as fear, depression, anxiety or stress. Coping behaviors, like resiliency and other ways of adapting to the pandemic, including religious, spiritual and community-oriented approaches highlighted experiences with stringent infection prevention and control measures to contain COVID-19 in the Philippines. The COVID-19 pandemic brought severe challenges to mental and psychosocial health in the Philippines. The literature focused mostly on healthcare workers and educators/learners, and moderate-to-severe mental health outcomes in these groups. There is a need to expand studies to other sociodemographic groups and communities across the Philippines. Future work stands to benefit from more in-depth qualitative, mixed methods, longitudinal and representative quantitative research in LMICs following this pandemic. Literature reviews remain important to synthesize post-pandemic experiences by providing context for future studies and health practice in the Philippines and other LMICs.

## Graphical abstract

**Figure:**
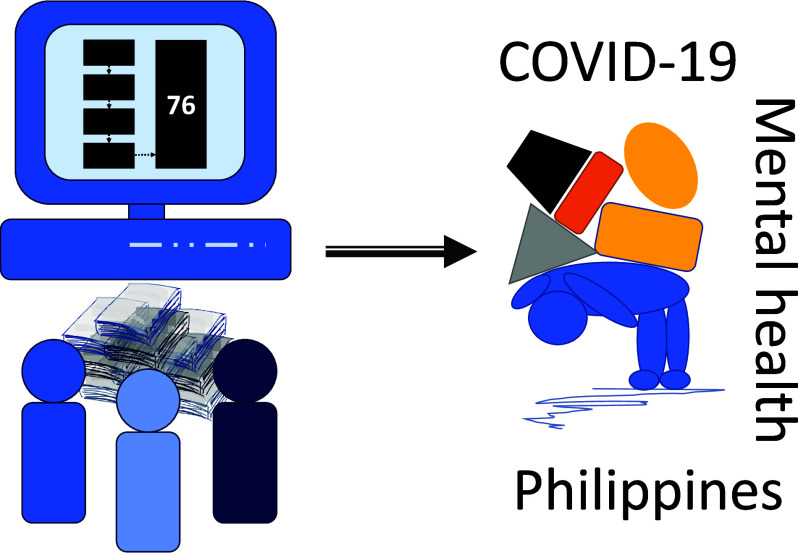

## Impact statement

This scoping review of research literature found and described the emerging evidence base outlining comprehensive effects of the COVID-19 pandemic on mental and psychosocial health in the Philippines. This work addressed existing knowledge gaps by bringing light to literature that looked at concrete health outcomes such as anxiety and depression, as well as highlighting some of the daily stressors facing the population in an LMIC setting such as the Philippines. Specifically, this article reflects the literature that was published during the first 2 years of the pandemic and contributes substantially to providing an overview of what research was done during this time. This article’s discussion anchors its findings in the country’s local context. This body of literature largely documented the mental and psychosocial health impacts specifically on educators, learners and health workers in the Philippines. It also highlighted remaining areas of focus and opportunities for learning, including providing guidance for future literature reviews, qualitative and quantitative research studies, public health practice and policy efforts relevant to the Philippines and other LMICs.

## Introduction

The Coronavirus disease of 2019 (COVID-19) pandemic disrupted lives globally, leaving millions affected, but still its impact on larger population health remains unknown (WHO, 2020a). Since its emergence, the world has gained tremendous insights into the pandemic’s consequences (CDC, 2020); however, knowledge is still lacking about its ripple effects on global mental health, especially in LMICs. Given prevailing research focus on high-income countries in the Americas and Europe, and persisting systematic and inequitable health system challenges, including less support to global mental health regionally (Sharma and Razzaque, 2017), LMICs in Southeast Asia remained at great risk for the pandemic’s effects on population health (Chookajorn et al., 2021). In May 2023, WHO declared an end to COVID-19 as a global health emergency, approaching 766 million cumulative cases and nearly 7 million deaths worldwide, of which more than one-third of all cases, and almost one-fifth of all deaths were in South Asia and the Western Pacific (WHO, 2023).

LMICs face the heaviest costs to global mental health (Rathod et al., 2017). The Global Disease Burden Index has demonstrated enormous costs resulting from mental disorders like major depressive and anxiety disorders, estimating costs around 125 million global disability-adjusted life years (Global Burden of Disease, 2020). Most of the world’s population reside in the 153 LMICs, and more than four out of five of all those facing mental health disorders live in these settings. Already predicted as a leading cause of disease burden (Mathers and Loncar, 2006), a long road remains ahead to adequately address projected mental and psychosocial health needs in LMICs. Exacerbated by far-reaching and lingering impacts of the pandemic, pressures on clinical mental health outcomes (e.g., severe psychiatric outcomes), and on broader psychosocial health (e.g., social, spiritual and emotional dimensions of human health and wellbeing) (Martikainen et al., 2002), the pandemic’s effects on global mental health in LMICs remains unclear.

As a significant contributor to the global health workforce, and an LMIC facing systematic health challenges with a layered colonial history, most relevantly including other nations’ long-term administration of local health matters, the Philippines provides important grounds for global mental health research on the ripple effects of the COVID-19 pandemic. Comprised of around 7,641 islands (Embassy of the Republic of the Philippines, Washington DC, USA, n.d.), a high population density, elevated levels of poverty and malnutrition and disruptions to health systems due to natural disasters and conflicts put the Philippines at risk for virological impacts and wider mental and psychosocial health consequences resulting from the COVID-19 pandemic (Lopez, 2020; World Bank, 2021a, 2021b).

Prior to COVID-19, the Philippines was already facing one of the highest rates of mental disorders in the Western Pacific, with an estimated 15.4 million Filipinos experiencing depression, 1 million living with schizophrenia, 15.3 million facing substance use disorders, and 877,000 losing their lives to suicide annually (Maravilla and Tan, 2021). Not only did the pandemic exacerbate severe health disorders, mental health outcomes such as anxiety and stress, and psychosocial health were also negatively affected. National government underscored the importance of mental health during the COVID-19 pandemic, but simultaneously noted the dearth of relevant resources (Daquioag, n.d.). As elsewhere, compromised policy and implementation focus on mental health has led to less availability of data monitoring and research in-country (Toquero, 2021). The Philippines passed the landmark Mental Health Act legislation in 2018 (Lally et al., 2019a); however, implementation barriers to seeking mental health and psychosocial support remain in-country (Tanaka et al., 2018). This includes underfunded programs, limited health financing and lack of mental health professionals with about 500 psychiatrists, 4,200 guidance counselors, 1,600 psychologists, (Aruta et al., 2022c) and 516 psychiatric nurses (WHO, 2020b) registered, but not necessarily in practice, to serve approximately 110 million people (Philippine Statistics Authority, 2021). Additionally, stigma, notably self-stigma and social stigma, present as substantial barriers to health-seeking behavior within mental health (Martinez et al., 2020). Economic constraints have affected people’s access to mental healthcare (Lally et al., 2019b), including historically low levels (5%) of healthcare expenditures devoted to mental health (Maravilla and Tan, 2021). While national funding levels devoted to mental health increased substantially following the pandemic (Senate of the Philippines 19th Congress, 2023), barriers remain, including elevated emigration rates of health professionals to other countries due to factors such as burnout and stress (Alibudbud, 2023) affecting the demand for training and recruitment of clinical, public health and social workers for local health needs (Lally et al., 2019b). Non-clinical aspects of psychosocial health are less emphasized or left out of mental health policy discussions.

During the early parts of the pandemic, humanitarian workers found little contact between communities and the mental health system/services in the Philippines (Okay Lang Ba Ang Mga Bata? 2021). Stigma was also found to be an important source of social exclusion in communities (Kahambing and Edilo, 2020). The Philippines maintained one of the strictest containment measures globally during the COVID-19 pandemic (Mathieu et al., 2020), including various levels of community level-quarantine approaches. Imposing acute and long-lasting population control interventions brought along heavy societal costs (Bayani and Tan, 2021), including unemployment, and there are standing questions about how strict lockdowns, quarantine, travel restrictions and isolation measures affected longer-term mental and psychosocial health. Before the lockdown, almost three quarters of children reported using health centers, but usage dropped to less than half during lockdown (World Bank, 2021a). Concerns regarding elevated levels of violence and abuse affecting communities increased during this health crisis. The ongoing pressures from natural disasters, on top of the COVID-19 pandemic had ramifications that make the Philippines an important setting for global research of mental and psychosocial health during and following the COVID-19 pandemic, but currently, there are limited data.

To mitigate suboptimal health trends over time resulting from this pandemic in LMICs, there is a need to go beyond the biomedical consequences of virological pathogenicity and address unanswered questions about the state of people’s mental and psychosocial health following the COVID-19 pandemic in countries like the Philippines.

### Study objective

This study reviewed public health literature from 2020 to mid-2022 to examine how the COVID-19 pandemic affected mental and psychosocial health in the Philippines.

## Methods

### PRISMA-ScR, search strategy, inclusion criteria, data bases and data charting

This review used the Joanna Briggs Institute methodology, Preferred Reporting Items for Systematic Reviews and Meta-Analyses Extension for Scoping Reviews (PRISMA-ScR), and the Population-Concept-Context (PCC) question model (Tricco et al., 2018; Table 1). This method enabled a broad examination of emerging literature, including indications of volume, and a topical overview of the literature surrounding the COVID-19 pandemic and mental and psychosocial health in the Philippines (Munn et al., 2018).

**Table 1.** Overview of Population-Concept-Context (PCC)-elements (University of South Australia, 2022) relevant to the search strategy of the larger review study of three Southeast Asian countries that this Philippines study was part of.

PCC element	Definition (per JBI Reviewer’s Manual Ch. 11)	Applications to this study
Population	“Important characteristics of participants, including age and other qualifying criteria” (11.2.4) You may not need to include this element unless your question focuses on a specific condition or cohort	General community members, health workers (clinical and non-clinical), educators/learners
Concept	“The core concept examined by the scoping review should be clearly articulated to guide the scope and breadth of the inquiry. This may include details that pertain to elements that would be detailed in a standard systematic review, such as the ‘interventions’ and/or ‘phenomena of interest’ and/or ‘outcomes’” (11.2.4)	Mental health, psychosocial health, COVID-19 disease and pandemic control measures
Context	“May include… cultural factors such as geographic location and/or specific racial or gender-based interests. In some cases, context may also encompass details about the specific setting”	Low and middle-income country settings, Southeast Asia, the Philippines (Indonesia and Vietnam reviews are ongoing and reported in separate papers)

**Figure 1.**
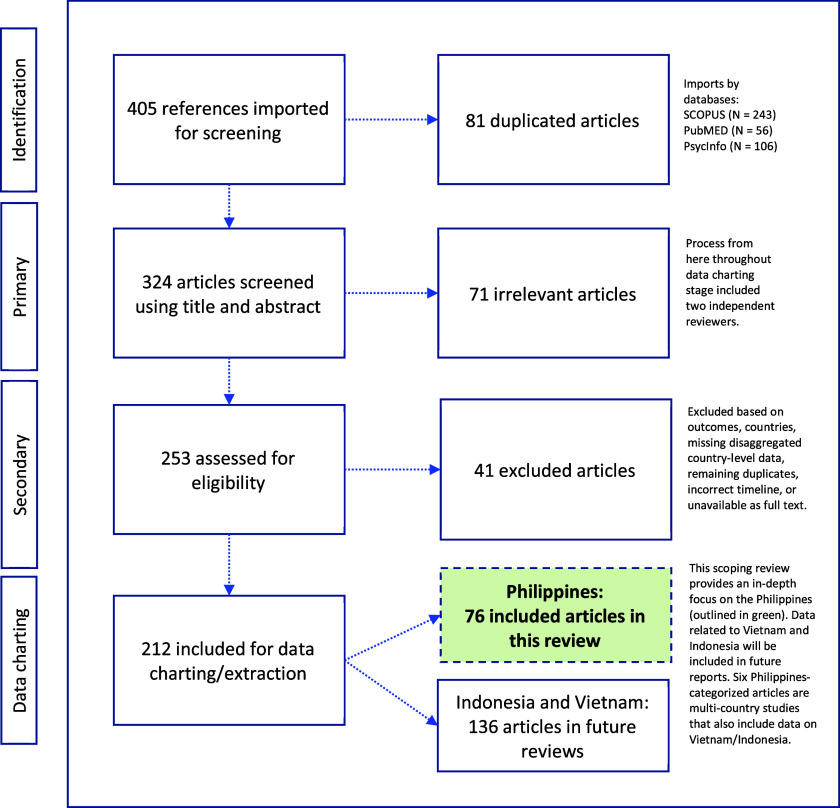
PRISMA-ScR with 76 identified articles pertinent to examining the effects of the COVID-19 pandemic on mental and psychosocial health in the Philippines.

In consultation with Columbia University, Augustus C. Long Health Sciences Library Informationists, the team followed a systematic strategy for developing, testing and finalizing Boolean search string for this literature review. Three conceptual categories operationalized the search string: (1) COVID-19 cases, deaths and related public health measures (e.g., quarantine, isolation); (2) mental and psychosocial health outcomes (e.g., depression, stigma, coping, services) and (3) select LMICs in Southeast Asia (i.e., Indonesia, the Philippines and Vietnam). While this overarching search strategy included three LMICs in Southeast Asia, this paper reports solely on the Philippines given the need for an in-depth focus about this country’s emerging evidence base (Figure 1). Findings from reviews about Indonesia and Vietnam are forthcoming in separate papers. The study country, the Philippines, is a highly diverse archipelagic nation with a population around 110 million, with communities of diverse social, cultural and historical backgrounds.

The search strategy was employed against three international databases recommended by university researchers and informationists to identify peer-reviewed literature: PsycInfo, PubMed and SCOPUS (see Appendix). The team performed hand searches and used PubMed search hedges (i.e., pre-defined search strings), MESH terms and key words, as appropriate. Prior to developing the final search string, the research team ran test rounds, broadly checking for specificity and sensitivity of preliminary search results. This review took place and included articles in English from January of 2020 through June 2022, and topics including, but not limited to different population groups, settings, and interventions (e.g., services, programs, policies) in the Philippines.

Following the identification of relevant articles and importation into COVIDENCE software, (COVIDENCE, 2023), two independent reviewers conducted primary screening of titles/abstracts, and secondary screening of full-text documents facilitating a blind screening process using COVIDENCE. Following article screening, two separate reviewers completed data charting, extracting relevant data from articles. Two independent reviewers analyzed and extracted data using a template and reviewed aggregated data, performed data cleaning, and built consensus around findings from this extraction process. The team resolved any differences or disagreements using internal conversations, and discussion with third parties, as needed. The team built an impression of the coverage, volume and content of available literature. This included reviewing aspects of completeness (e.g., findings), data quality (e.g., study design), representation (e.g., study population), ethics (e.g., institutional approvals) and biases (e.g., study design). No ethical approval was needed for this scoping review of openly available research literature.

## Results

### PRISMA-ScR findings

This literature review identified 405 publications across PubMed (*N* = 56), PsycInfo (*N* = 106) and SCOPUS (*N* = 243). After automated duplicate removal by COVIDENCE, (*N* = 81 articles), 324 articles advanced to primary screening with two reviewers, who classified 71 documents as irrelevant. Secondary screening with two reviewers followed, who assessed 253 full-text documents and excluded an additional 41 articles because they included the wrong health outcome(s), incorrect country, were multi-country studies about relevant countries but with insufficient disaggregated information on the country level, were remaining duplicates that had not been removed by the automated process, wrong timeline, or were unavailable in full-text versions. From the remaining 212 relevant articles about Southeast Asian LMICs, 76 publications were about the Philippines (including six articles also about Indonesia and Vietnam) (Figure 1, in green).

**Figure 2.**
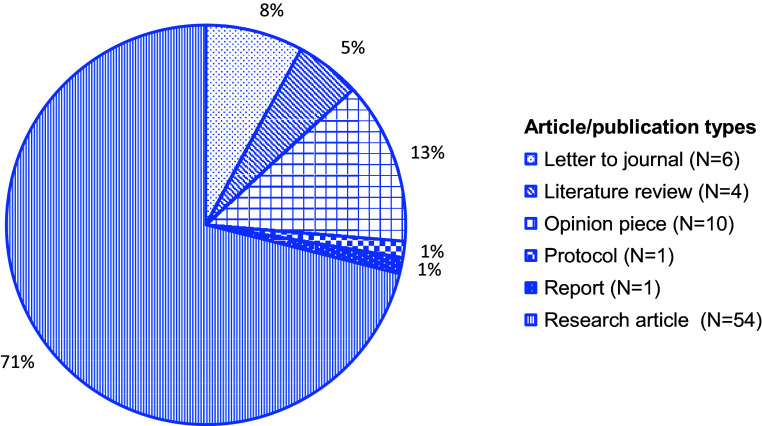
A pie chart showing the categorization of articles (*N* = 76) into publication types (%, *N*) in this scoping review examining the COVID-19 pandemic’s effects on mental and psychosocial health in the Philippines.

**Figure 3.**
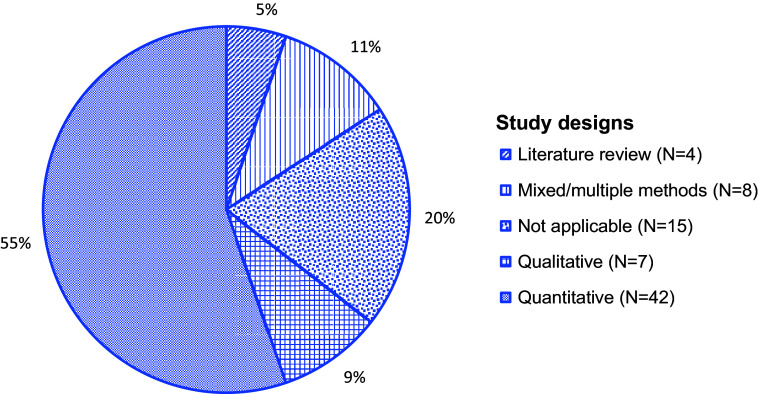
A pie chart showing the categorization of articles (*N* = 76) and their study designs (%, *N*), where applicable, in this scoping review examining the COVID-19 pandemic’s effects on mental and psychosocial health in the Philippines.

This study found 76 articles addressing aspects of the COVID-19 pandemic and mental and psychosocial health in the Philippines. Most publications were descriptive studies or articles commenting on interventions (Supplemental Table). This review saw varying degrees of alignment between commentaries/letters/opinion pieces and research papers, including agreement on mental health consequences of crisis-driven online learning (Baloran, 2020; Alibudbud, 2021), but calls to focus on the older adult populations were not met in the research literature (Buenaventura et al., 2020). While this scoping review did not undertake a comprehensive data quality evaluation, the identified literature consisted of low to very high-quality publications, as understood by articles providing sufficient and systematic information and data where needed. A total of 54 were research articles and 10 were opinion pieces (Figure 2). More than half (55%) were published in 2021, followed by 2022 (26%), and 2020 (18%). While most (81%) articles were published between 2021–2022, about two-thirds of all articles (67%) reported using data from 2020, early in the pandemic. Many research articles used quantitative study designs (*N* = 42, 55%), followed by eight articles (11%) noting multiple or mixed methods, seven (9%) studies using qualitative designs, and four (5%) performing literature reviews (Figure 3). Quantitative studies using online survey methodologies, addressing healthcare staff/trainees and educators and learners, largely noted health outcomes such as stress, anxiety and fear. Of 54 research articles and one study protocol, 50 reported using non-probabilistic sampling methods (e.g., snowball sampling), and five reported probabilistic sampling methods (e.g., stratified sampling). Most research studies collected or had plans for collecting primary data. About half, or 20 of 46, of all research studies reported having local Internal Review Board (IRB)/similar approval in the Philippines, eight had local plus an out-of-country IRB/similar approval, seven had out-of-country approvals (Canada, Hong Kong, Poland, Republic of Korea, Singapore, United States of America), and 11 noted using other types of study approvals.

### Mental and psychosocial health

Topically, this body of literature was largely about anxiety (45 studies), stress (44 studies) and depression (30 studies). Severe outcomes such as posttraumatic stress disorder (PTSD), and other psychosocial aspects such as quality of life were reported less frequently (Supplemental Table). A study of adults largely under quarantine across different regions in the Philippines reported severe anxiety (13.84%), moderately severe depression (21.73%) and severe depression (9.08%) (Bernardo et al., 2020). During enhanced community quarantine, 1,879 participants shared moderate-to-severe anxiety (28.8%), moderate-to-severe stress levels (13.4%) and moderate-to-severe depression (16.9%) (Tee et al., 2020b). Younger age groups, including children and adolescents (12–21.4 years), single persons, and people without children experienced higher stress, anxiety and depression (Tee et al., 2020b). A country-comparison of adults (wave 1: *N* = 1,041, wave 2: *N* = 1,003) across Belgium, Canada, England, Hong Kong, New Zealand, Switzerland and the United States of America, showed that adults in the Philippines experienced some of the highest levels of Generalized Anxiety Disorder and Major Depressive Episode (Généreux et al., 2021).

There was limited research on stigma in the Philippines, except findings of generalized stigma discussions in LMICs and other settings (Javed et al., 2021; Nashwan et al., 2022). Psychological distress affected quality of life, mediated by financial resources, feeling safe at home and trusting public institutions in the Philippines (Aruta et al., 2022a). Health workers reported coping behaviors (Labrague, 2022), such as personal resilience (Labrague and De Los Santos, 2020). There was a moderate impact on the quality of life of teachers almost 6 months after the nationwide lockdown (Rabacal et al., 2020). Spirituality (Bangalan, 2022; Cordero, 2022) and religion (Del Castillo et al., 2020) emerged as recurring themes in effective coping and stress management. However, researchers brought up unmet spiritual needs among advanced-age populations in the Philippines (Buenaventura et al., 2020).

Researchers used different theories, scales and frameworks to guide their research studies during the COVID-19 pandemic. Commonly found among quantitative studies included standardized scales, such as the Depression, Anxiety and Stress Scales 21-items (DASS-21), the Brief Resilience Scale (BRS) and Impact of Events Scale-Revised (IES-R) (Table 2).

**Table 2.** Summarized table of highlighted findings from this scoping review.

Characteristics		*N*	%
Multi-country versus single-country study	Multiple-country study (including the Philippines)	18	33
Research articles/studies (*N* = 54)	Single-country study (Philippines only)	36	67
Geographically specified	Luzon	10	28
areas in the Philippines in	Visayas	9	25
single-country research articles/studies (*N* = 36)	Mindanao	1	3
	National/multiple regions	3	8
	Other (including not specified)	13	36
Common scales in research articles/protocol (*N* = 55)	Depression, Anxiety, and Stress Scales 21-items (DASS-21)	10	18
	Brief Resilience Scale (BRS)	7	13
	Impact of Events Scale-Revised (IES-R)	5	9
	Patient Health Questionnaire 9-items/4-items	4	7
	Fear of COVID-19 Scale (FCV-19S)	4	7
	Brief Coping Orientation to Problems Experienced Inventory Scale (Brief-COPE) 28-items	3	5
	Other	33	60
Respondent size from all research articles/studies in the Philippines (range)	Low, medium, and large respondent sizes (interval of minimum and maximum)	6 to 2,119	–
Population segment in all	Health professionals	23	30
publications (*N* = 76)	Educators/learners	22	29
	Adults	12	16
	Adolescents and/or children	5	7
	Disaster-affected persons	3	4
	Not specified/general population/other	11	14

*Note:* See Supplemental Table for a complete list of identified publications.

About less than half (*N* = 36) of research studies analyzed focused exclusively on the Philippines, with an additional 18 research articles including cross-country comparisons, with countries such as the United States of America, China and Vietnam (Table 2). Small, medium and large-scale studies ranged from a minimum of six, to a maximum of 2,119 respondents across both qualitative and quantitative studies (Table 2).

### Health professionals, educators/learners and other groups

Health professionals’ mental and psychosocial health was negatively affected by the pandemic. Frontline nurses from different parts of the country with moderate levels of personal resilience and social and organizational support, experienced dysfunctional levels of anxiety (*N* = 123, 37.8%) (Labrague and De Los Santos, 2020). Compassion fatigue, fear and coronaphobia, the “excessive triggered response of fear of contracting the virus… causing marked impairment in daily life functioning” (Arora et al., 2020), specifically challenged frontline hospital and public health nurses (Labrague and De Los Santos, 2021a, 2021b, 2021c), and physicians (*N* = 53, 89.83%) noted increased levels of burnout (Tan-Lim et al., 2022). In addition to direct challenges to their mental and psychosocial health, health professionals also managed the effects on their patients. A patient population (*N* = 512) with Systemic Lupus and Rheumatoid Arthritis reported moderate to severe anxiety (38.7%) and moderate to severe depression (27%) (Tee et al., 2020a). A tuberculosis (TB) cohort study faced operational follow-up issues during the pandemic, when participants expressed fears regarding feeling more at risk for COVID-19 than their TB (Ferrer et al., 2021).

Anxiety was reported across educator/learner populations with varying levels, including 62.64% (332/530) of students being worried about food and financial resources, and about half noted avoiding in-person socialization and group meetings during lockdown in southern Philippines (Baloran, 2020). A high school student population reported moderate levels of cognitive anxiety related to COVID-19, and educators and learners in the Philippines generally encountered challenges with internet connection, limited technological resources and training (Dy et al., 2021). Students (e.g., nursing, medical, college) reported isolation, boredom, and sadness following abrupt educational changes and lockdown (Barrot et al., 2021). Information technology overload was another challenge of online learning during the pandemic (Alibudbud, 2021). Persons connected to university networks in the Philippines saw elevated levels of depression, anxiety and stress compared to their counterparts in China (Tee et al., 2021).

Broadly, female participants mostly contributed to these data, with exceptions such as a study about burnout among gastroenterologists in Southeast Asia (Ong et al., 2021). Largely, the focus remained on health professionals (*N* = 23) and educators/learners (*N* = 22), with limited mention of other groups, like adults (*N* = 12), children (*N* = 3), disaster-affected populations (*N* = 2), people with disabilities (*N* = 2) and others (Supplemental Table). While there were no studies specifically on advanced-age population groups, researchers called attention to the impact on this population in the Philippines (Buenaventura et al., 2020).

### Co-occurrence of natural disasters

Substantial impacts of co-occurring natural disasters continued to affect mental and psychosocial health in the Philippines. Typhoon season severely affected people who had to manage the multiplicity of challenges to their health during this pandemic (Rocha et al., 2021; Izumi and Shaw, 2022). While disaster-related challenges persisted, researchers found learning opportunities related to coping during multiple disaster(s). In some instances, they reported positive learning effects following typhoons during the COVID-19 pandemic (Cueto and Agaton, 2021).

### Public health and social interventions

According to the Government Stringency Index measuring the strictness of global policy responses during the pandemic (Mathieu et al., 2020) reflected in school closures, workplace closures and travel bans, the Philippines implemented strict measures throughout different parts of the pandemic (Lee et al., 2021). Enhanced Community Quarantine (ECQ)s caused movement restrictions and business operations limitations for all, except essential workers (Teng-Calleja et al., 2020). From 2020 to 2022, communities experienced several rounds of ECQs. Strict measures led to lockdown fatigue in the Philippines (Egcas et al., 2021) and researchers noted that higher levels of depression in-country might be related to intervention stringency compared to other countries (Lee et al., 2021).

There were numerous ways of adapting to public health and social measures during the pandemic in the Philippines. A study from 25 different industries across the Philippines found individual ways of coping, including task-focused coping, stress management, social coping and other strategies (Teng-Calleja et al., 2020). Additionally, the study found organizational level-coping strategies like flexible work arrangements, mental health and well-being programs, physical health and safety measures. While adapting to the crisis, healthcare workers combined established and new strategies in teaching and learning during medical training (Chua et al., 2020) and engaged preventive interventions to mitigate physician burnout (Tan-Lim et al., 2022).

Health authorities in the Philippines offered virtual health services to the public (Leochico et al., 2020), including the National Center for Mental Health Crisis Hotline and Project HopeLine, a COVID-19 suicide prevention and crisis helpline (Corpuz, 2020). However, according to a literature review, the Philippines was one of several Southeast Asian countries with limited telepsychiatry services, including low accessibility for children and elderly populations stemming from poor internet connection and inability to access equipment for telepsychiatry services (Narvaez, 2022). Others found while undergoing training, many health practitioners had engaged in teleneurology services (Pagaling et al., 2022). A mixed-methods evaluation highlighted stakeholder support for teletherapy as a useful service model and in the case of children with neurodevelopmental disorders, teletherapy proved effective (Eguia and Capio, 2022). However, there was scarcity of child-centered interventions indicated by a worsening of the state of mental well-being of children in the Philippines (Malolos et al., 2021).

Interventions noted emphasized social cohesion and community work. Researchers underscored the importance of considering collectivist cultures, including aspects of social support (Hechanova et al., 2021). Social support plays an important part of psychological resilience following disasters, and researchers developed an asynchronous (online) peer support program, which aimed to improve psychological well-being, resilience, adaptive coping and decrease depression symptoms (Hechanova et al., 2021). Online religious services were available to mitigate negative impacts on mental and psychosocial health through online-based masses, community prayers and spiritual collections (Del Castillo et al., 2020). Other interventions included community pantries to benefit children’s mental health (Kahambing, 2021) and home gardening to promote well-being, and happiness by mitigating stress and anxiety (Sunga and Advincula, 2021).

## Discussion

The COVID-19 pandemic presented severe challenges to mental and psychosocial health in the Philippines. This review found mostly moderate-to-severe levels of health outcomes such as depression, anxiety and stress, especially among health workers and educators/learners. In multi-country studies, people from the Philippines stood out as the most affected by poor mental health outcomes. This corroborates with other findings, making the case for investing in research, resources and interventions addressing mental health and related challenges in the Philippines moving forward (WHO, 2021).

Health workers in the Philippines were negatively affected during the pandemic (Rillera Marzo et al., 2021). This also applies to their colleagues who emigrated to other countries. In the United States of America, health workers from the Philippines, who formed a large part of the frontline pandemic workforce, faced threats and discrimination (Sangalang, 2022). Home care workers remained both invisible and exposed to racial inequities (Nasol and Francisco-Menchavez, 2021). Health workers in the Philippines affected by low pay were also affected by a deployment ban to other countries during the pandemic, highlighting how intricately linked the global health workforce remains across borders (PUBLiCUS, 2020).

The Philippines’ geographical proximity to regular and intense tectonic plate activity elevates the population’s risk for grave public health consequences of natural disasters, including stressors on mental health. On average, the country experiences 10–25 natural disasters, 20 tropical cyclones and an estimated 900 earthquakes each year (Bollettino et al., 2018; Guha-Sapir et al., 2018). And these numbers are likely to change because of the ongoing changes in climate. Volcanic eruptions and flooding events lead to frequent forced displacement events. These forces of nature did not rest during the pandemic (Rocha et al., 2021). Separating out mental health outcomes (e.g., depression) from these substantial and multiple daily stressors continuously experienced by communities in the Philippines is not feasible in practice. Research that goes beyond linear understanding of mental health outcomes is sorely needed. Objectives and methods that comprehensively address how COVID-19 directly and indirectly interact with everyday stressors for different populations will help generate a more complete understanding of the impacts of this pandemic crisis on mental and psychosocial health in the Philippines.

Tools with online operational ease during mass containment benefitted researchers amid the pandemic. Most quantitative studies used online data collection early in the pandemic and standardized scales, like DASS-21 and/or IES-R (Table 2). While operationally beneficial, research challenges remain as standardized tools in global mental health are heavily skewed toward Western-centric constructs, mental models and epistemologies. To translate survey instruments to Filipino language, Aruta et al. (2022a) used double-translation and the reconciliation method, and enlisted experts to identify areas of linguistic, psychological and cultural equivalence. Another study by Aruta et al. (2022b) compared structural validity of three measurement models for mental health in their survey of typhoon survivors.

However, some research tools found in this review may not have been adequately validated in the context of the Philippines. When applied without adequate contextualization, research findings may highlight only Western-skewed constructs. A preference toward Western constructs may be the result of a landscape shaped by European and US colonization, anchored in Western ways of understanding mental health. Consequences that follow may include overlooking indigenous and local aspects of health and wellbeing in the Philippines, especially dimensions important to mental health. Without proper consideration and contextualization, the Western European and North American-based understanding of health underpinning standardized scales can potentially lead to misrepresentative data and inaccurate portrayals of people’s lived health experiences in other settings. This country’s diverse sociocultural and historical background, with numerous languages and dialects, mixed composition of highly urbanized, and rural, provincial settings, indigenous and non-indigenous populations, and a highly globally mobile population, adds nuanced layers to the health priorities and resources in-country. An elevated number of people living below the poverty line (18.1%), clan politics, intercommunal tensions and armed conflict additionally contribute to the need for a multi-layered and deeply comprehensive public health approach (ACAPS, 2021; Philippine Statistics Authority, 2022). Efforts to indigenize/decolonize Filipino psychotherapeutic approaches include the rise of *Sikolohiyang Pilipino* (Filipino Psychology) (Tuliao et al., 2020), questioning the applicability of Western psychological concepts with Filipinos while also understanding Filipino thought and experience from a Filipino perspective. Currently, the field seems to be moving toward integrating local and global practices to best suit the populations in the Philippines.

The public coped in multiple ways during COVID-19 using a variety of mitigative interventions. Many sought support in faith-based organizations, and used online, group-based ways to find social support (Del Castillo et al., 2020; Hechanova et al., 2021). This highlights the importance and value of locally contextualized, collectivist and community-driven approaches to intervention planning that can benefit diverse populations in the Philippines.

The focus on healthcare workers in clinical settings, and learners/educators in academic settings here, reflect ongoing research interests, but may also reflect the practical nature and accessibility to these research groups early in the pandemic. It remains imperative to continue research efforts with these groups, in addition to expanding focus to other demographic groups. The lived experiences of health professionals and educators/learners substantially differ from other groups, especially given the organizational structures and unique resources associated with, for example, hospitals and universities. This review found little research on younger populations. In the Philippines, children, and adolescents, as well as young adults, went through almost 2 years of online education and could only return fully in-person to their classrooms at the end of 2022 (Gutierrez, 2022). More research is urgently needed at the intersection of the educational and health sectors to identify the health effects on children and adolescents during and following COVID-19. The pandemic negatively affected the mental health of children generally. Children were exposed to increased incidence for child labor, domestic violence and child abuse during this health crisis, often associated with challenges such as job and income loss, and strict population control interventions (Malolos et al., 2021). Additionally, populations may be at risk for poor mental health outcomes due to increasing occurrences of severe climatic challenges in the Philippines (Guinto et al., 2021), including younger generations experiencing climate change anxiety (Reyes et al., 2023).

A higher percentage of people in the healthcare sector or those completing surveys identify as female, which may have contributed to the large representation of females in these data. This scoping review found mostly quantitative research studies that used non-probabilistic sampling methods for online primary data collection during the early phase of the pandemic. This may reflect practical difficulties in using probabilistic sampling methods during major containment measures in the Philippines. Similarly, constraints prevented researchers from conducting in-person qualitative work during these phases of the pandemic. Given that not all people had access to internet or information technology equipment, the voices of communities without these resources remain underrepresented in the literature. More research is urgently needed among these groups. Most research studies in this review had ethical approvals with organizations in the Philippines, which represents aspects of local ownership for these research studies.

### Limitations

Due to the recency of the COVID-19 pandemic, studies on its long-term mental health effects are limited across LMICs and other income settings. Researchers contributing to this body of literature, including authors of this paper, were limited by urgency and to online data collection during the height of the pandemic. While English is one of the official languages in the Philippines, the use of English in this literature review and in the larger academic publication system steers findings toward a Western-focused and high-income country-direction of academic knowledge production and generation. This may introduce language, cultural and related biases into the evidence, including those reported herein. These findings and their interpretation share constraints with the study team’s capacity, databases, search strings, and study timeline used here, which does not include articles post-June 2022 or a local database. However, data following the conclusion of this literature review share similar findings, such as how public health workers experienced mental health exhaustion and fatigue as they delivered non-COVID-19 health services after the first wave of the pandemic (Maravilla et al., 2023). Others have also pointed to reduced barriers to mental health care and stigma, and increased use of telehealth and its positive impacts in the Philippines (Bollettino et al., 2023). Recent qualitative work has also reported stress, mental health exhaustion, fear of infection, work overload, pressure to learn new technology, and webinar fatigue among public health workers in the Philippines (Maravilla et al., 2023).

### Future research and perspectives

Future research stands to substantially benefit from more in-depth qualitative, mixed methods and longitudinal studies that can help contextualize and bring cultural understanding to mental and psychosocial health in the Philippines. Representative quantitative studies across different regions in the Philippines would help explore generalizability of health findings across this highly diverse country following the pandemic. Future systematic reviews/meta-analyses on concrete health outcomes across specific groups in the country may add value to our understanding of specific clinical effects of the pandemic but should not be interpreted in a vacuum. To expand on the existing literature, future research should include other population groups, such as children, advanced-age adult populations, indigenous populations, people with disabilities, migrant workers, those who experience homelessness, those in geographically isolated areas and other disadvantaged populations. With recall bias now affecting our ability to accurately report the lived experiences from this pandemic, it becomes an urgent matter to prioritize public health research in LMICs like the Philippines moving forward.

## Conclusion

This scoping review identified an emerging evidence base underscoring that the COVID-19 pandemic brought severe challenges to mental and psychosocial health in the Philippines. This study found that published research literature was mostly focused on healthcare workers and educators/learners, and moderate-to-severe mental health outcomes (e.g., stress, anxiety, depression) across these groups. Future studies on the COVID-19 pandemic’s health consequences are essential and will benefit from more in-depth qualitative and quantitative research of different communities across the Philippines and other LMICs.

## Supporting information

Ocampo et al. supplementary material Ocampo et al. supplementary material

## Data Availability

The authors confirm that the data supporting the findings of this study are available within the article and/or its Supplementary Materials.

## Supplementary material

The supplementary material for this article can be found at http://doi.org/10.1017/gmh.2024.14.

## Appendix. Boolean search strings applied to this scoping review in mid-2022

### PsycInfo

(TI (“COVID-19” OR “SARS-CoV-2” OR “post-acute COVID-19 syndrome” OR “COVID-19 stress syndrome” OR “COVID-19 post-intensive care syndrome”) AND (“mental health” OR “mental health services” OR “mental health associations” OR “community mental health services” OR “community mental health centers” OR “mental health recovery” OR “psychiatry” OR “psychiatric nursing” OR “psychiatric rehabilitation” OR “health promotion” OR “psychosocial support systems” OR “social support”) AND (“the Philippines” OR “Vietnam” OR “Indonesia”)) OR (AB (“COVID-19” OR “SARS-CoV-2” OR “post-acute COVID-19 syndrome” OR “COVID-19 stress syndrome” OR “COVID-19 post-intensive care syndrome”) AND (“mental health” OR “mental health services” OR “mental health associations” OR “community mental health services” OR “community mental health centers” OR “mental health recovery” OR “psychiatry” OR “psychiatric nursing” OR “psychiatric rehabilitation” OR “health promotion” OR “psychosocial support systems” OR “social support”) AND (“the Philippines” OR “Vietnam” OR “Indonesia”))

### PubMed

(“COVID-19”[Title/Abstract] OR “SARS-CoV-2”[Title/Abstract] OR “post-acute COVID-19 syndrome”[Title/Abstract] OR “COVID-19 stress syndrome” [Title/Abstract] OR “COVID-19 post-intensive care syndrome”[Title/Abstract]) AND (“mental health”[Title/Abstract] OR “mental health services”[Title/Abstract] OR “mental health associations” [Title/Abstract] OR “community mental health services [Title/Abstract] OR “community mental health centers”[Title/Abstract] OR “mental health recovery”[Title/Abstract] OR “psychiatry”[Title/Abstract] OR “psychiatric nursing”[Title/Abstract] OR “psychiatric rehabilitation”[Title/Abstract] OR “health promotion”[Title/Abstract] OR “psychosocial support systems” [Title/Abstract] OR “social support”[Title/Abstract]) AND (“the Philippines” [Title/Abstract] OR “Vietnam”[Title/Abstract] OR “Indonesia”[Title/Abstract] “Indonesia”)

### SCOPUS

TITLE-ABS-KEY (“covid-19” OR “sars-cov-2” OR “post-acute covid-19 syndrome” OR “covid-19 stress syndrome” OR “covid-19 post-intensive care syndrome” AND “mental health” OR “mental health services” OR “mental health associations” OR “community mental health services” OR “community mental health centers” OR “mental health recovery” OR “psychiatry” OR “psychiatric nursing” OR “psychiatric rehabilitation” OR “health promotion” OR “psychosocial support systems” OR “social support” AND “the philippines” OR “vietnam” OR “indonesia”) AND PUBYEAR > 2019 AND PUBYEAR < 2023 AND PUBYEAR > 2019 AND PUBYEAR < 2023.
