# Supplementary material for: The COVID-19 pandemic’s effects on mental and psychosocial health in the Philippines: A scoping review
Source: Glob Ment Health (Camb). 2024 Feb 8;11:e27. doi: 10.1017/gmh.2024.14 (PMC10988143; doi:10.1017/gmh.2024.14)
Supplement: Ocampo et al. supplementary material [file S2054425124000141sup001.pdf]

Supplemental Table. An overview of 76 articles reflecting mental and psychosocial health during the COVID-19 pandemic in the Philippines.

Author, year (publication type)	Title	Study objective	Mental and psychosocial health topics and themes (outcomes and interventions, as applicable)	Data collection	Primary study population (N participants, if applicable) and setting (where provided)	Key findings
**Alibudbud 2021 (Opinion piece)**	**On online learning and mental health during the COVID-19 pandemic: Perspectives from the Philippines**	To discuss online learning and mental health during the COVID-19 pandemic in the Philippines, including to analyze what measures cognizant of the resources of a developing country are needed to mitigate the mental stresses from online learning including videoconferencing.	Reflections on negative mental health consequences of online learning during the pandemic.	Non-applicable (NA)	Educator/learner population (N=Not applicable (NA)) in an educational setting in the Philippines.	Generally, learning that considers the child´s mental health should take cognizance of the circumstances that children faced in their daily social environment. While there is evidence that specific actions contribute to better mental health among children, the outcomes are contingent on context. In the case of a developing country context, teaching children in the COVID-19 era requires the consideration of existing social inequalities and economic constraints to safeguard their mental health in the online learning environment. This study noted that many suggestions for developed settings that do not work in developing contexts.
**Alibudbud 2022 (Research article)**	**Googling depression and major depressive disorder after mental health legislation and during the COVID-19 pandemic in the Philippines: An infodemiological study**	To utilize Google Trends to explore Filipinos´ interest towards depression over time.	About depression, mental health law, stress, and mental health law.	Online search queries to collect primary data using for Google keywords (Google Trends).	Adult and general population (N=NA) in community setting in the Philippines.	Findings suggest increasing interest in understanding depression manifestations, etiology, diagnostic tests, and local context including available services, vernacular translations, and educational materials. Anxiety-related search queries were noted with depression as a mood and disorder. Overall, online public interest in depression in the Philippines increase with the MH law approval and decrease during the pandemic.

**Aruta et al 2022 (Research article)**	**The mediating role of psychological distress on the link between socio-ecological factors and quality of life of Filipino adults during COVID-19 crisis**	To determine how psychological distress operates as an underlying mechanism in the impact of socio-ecological factors on the quality of life of Filipino adults during coronavirus disease 2019 (COVID-19) crisis.	Addressed safety at home, trust in public institutions, and financial difficulties, psychological distress, anxiety, and quality of life during the early phase of COVID-19 community quarantine in the Philippines. Also brings up psychosocial support services, and relevant policymaking.	Survey using primary data using the Brief Symptoms Inventory (BSI), the 9-item My Life Today (MLT) scale, the National Resilience Scale, a Safety at home-single item question, and a financial difficulties single-item question.	Adult population (N=401) across community settings in the Philippines.	Findings indicate that psychological distress is a psychological mechanism that can partly explain why socio-ecological factors (i.e., safety at home, financial difficulties, and trust in institutions) impact the quality of life of Filipino adults during the COVID-19 crisis. People facing threats to home safety, financial instability, and distrust in public institutions in times of crisis are vulnerable to emotional symptoms, and reduced quality of life. These findings, therefore, provide evidence regarding the role of ecological factors in the development of and prevention from mental health concerns and diminished quality of life in times of great adversities such as the COVID-19 pandemic.

**Aruta et al 2022 (Research article)**	**Measurement and community antecedents of positive mental health among the survivors of typhoons Vamco and Goni during the COVID-19 crisis in the Philippines**	To examine the psychometric measurement and antecedents of positive mental health in people who concurrently experienced two disasters of different nature (i.e., typhoons and COVID-19 crisis), focusing on the survivors of typhoons Vamco and Goni that hit the Philippines in November 2020, during the COVID-19 pandemic.	Focused on positive mental health, individual resilience, community resilience, social responsibility, depression, anxiety, Post-Traumatic Stress Disorder, and psychological distress.	Survey to collect primary data using the 14-item Mental Health Continuum-Short Form, the 10-item Connor-Davidson Resilience Scale, the Conjoint Community Resilience Assessment Measure (CCRAM-10), and the 7-item Perceived Social Responsbility Scale (PSRS).	Disaster-affected population (N=447) in community settings in the Philippines.	Found support for the structural validity, criterion validity, and internal consistency of the MHC-SF items in assessing positive mental health in Filipinos. Findings indicate that MHC-SF is a valid and reliable tool in assessing EWB, SWB, and PWB of Filipinos in times of multiple disasters. Findings confirmed that social responsibility mediates the positive influence of community resilience on the three subscales of positive mental health among Filipinos within the contexts of extreme weather events occurring during the COVID-19 pandemic. Community resilience is (also) an important promoting factor of positive mental health in times of calamities. People tend to have better mental health when they perceive their local community as capable of with-standing disasters, and those who have a strong perception of social responsibility develop greater levels of emotional, social, and psychological well-being.

**Aung et al 2021 (Research article)**	**Community responses to COVID-19 pandemic first wave containment measures: a multinational study**	To explore community responses to COVID-19 containment measures in different countries and to synthesize a model.	Focused on loneliness, depression, anxiety, stress, and community response to COVID-19 containment measures. Also looked at health literacy, economic support, social services, and community engagement.	Survey and online group session to collect primary data using their own survey.	Health professionals; Other: Researchers (N=>70) in various educational settings across the Philippines, Indonesia, Japan, Austria, India, Malaysia, Myanmar, Sudan, Taiwan, Thailand, United States of America, and Vietnam.	A number of lessons can be learned from how the various countries responded during the first wave of the COVID-19 pandemic, although it is difficult to determine which specific intervention was the most effective. In terms of governments´ policy responses to the outbreak of COVID-19, several strategies were implemented simulteanously in the hope that one or more would be effective in containing the spread of the pandemic. In practice, the impact of the policies implemented were dependent upon the specific social and cultural determinents as presented in this study model. Emphasizes cultural-sensitivity, special needs, health beliefs and disease prevention behaviors.
**Balakrishnan et al 2022 (Literature review)**	**COVID-19 mental health prevalence and its risk factors in south east asia**	To synthesize the extant literature reporting the effects of COVID-19 pandemic on the psychological outcomes of people in Southeast Asia, and its risk factors.	Covered domestic abuse, suicide, poor mental health, stress, and depression.	Document review to collect secondary data. Did not use any scales, but listed in literature review, including: DASS-21, GAD-7, STAI, IES-R, HADS, PSS, ZAS, PHQ9, OLBI, and IWP-1.	Health professionals population and general adult population (N=NA), in several countries in Southeast Asia, including the Philippines.	The scoping review examined the impact of COVID-19 and its restrictions on the mental health of people in SEA countries between January 2020 and March 2021, and its associated risk factors. Elevated prevalence of adverse mental effects was noted in most of the countries as the pandemic progressed over time, with Malaysia and the Philippines reporting higher prevalence rates.

Baloran 2020 (Research article)	**Knowledge, attitudes, anxiety, and coping strategies of students during COVID-19 pandemic**	To assess knowledge, attitudes, anxiety, and personal coping strategies of college students during the COVID-19 pandemic in Southern Philippines.	Discusses knowledge, attitudes, anxiety, and coping strategies, and interventions such as stopping school, online blended learning approach, extension of the opening of classes for the next semester, lockdown.	Online survey (cross-sectional study) to collect primary data, no standardized scale, with questions adapted from other works.	Educator/learner population: College students (N=530) in educational settings: two local private colleges in province of the Davao del Sur, Mindanao, in the Philippines.	The COVID-19 pandemic posed significant concerns among students, especially among communities in the Southern Philippines, but were aware of the COVID-19 pandemic and possessed sufficient knowledge about the COVID-19 pandemic. Majority of the students displayed anxiety during the entire period of lockdown. 62.64% (332/530) were worried about food and financial resources, and about 54% to 56% of the students avoid social contact, large meetings, and gatherings. A considerable percentage of some students showed distrust towards the COVID-19 vaccine. In the context of education, students were reluctant toward the implementation of online-blended learning approach due to technological and financial constraints. Students were responsive and satisfied with the government´s initiatives to limit the spread of infection, though there was increased anxiety felt by students regarding getting the COVID-19 infection.
Bangalan 2022 (Research article)	**Mental health and protective strategies among community-based health workers in region 3, Philippines during COVID-19 pandemic**	To determine the prevalence of mental health outcomes among community-based health workers during the COVID-19 pandemic and examined the association of protective strategies with symptoms of mental health outcomes.	Focused on stress, anxiety, and depression and association of protective strategies (e.g., breathing exercise, social connection, and religious or spiritual practices) and the symptoms of mental health outcomes.	Survey to collect secondary data using Depression, Anxiety, and Stress Scale (DASS-21).	Health professionals population (N=324) in community setting in Central Luzon (Region 3) in the Philippines.	This study showed that community-based health workers had considerable rates of symptoms of stress (10%), anxiety (26%), and depression (18%). The engagement in regular spiritual/religious activities was found to be associated with symptoms of stress. Those individuals who regularly engage in religious/spiritual practices were less likely to report symptoms of stress, yet, this association should be further explored.

**Barrot et al 2021 (Research article)**	**Students´ online learning challenges during the pandemic and how they cope with them: The case of the Philippines**	To investigate students´ online learning experience in higher education within the context of the pandemic, by specifically identifying the extent of challenges that students experienced, how the COVID-19 pandemic impacted their online learning experience, and the coping strategies that they used to confront these challenges.	Adrdressed anxiety, boredom, sadness, isolation, quality of and challenges to online learning and student mental health.	Survey/questionnaire and focus group discussions to collect primary data using a new scale.	Educator/learner population: students (N=200) in an educational setting in the Philippines.	Findings revealed that the online learning challenges of students varied in terms of type and extent. Their greatest challenge was linked to their learning environment at home, while their least challenge was technological literacy and competency. Based on the students´ responses, their challenges were also found to be aggravated by the pandemic, especially in terms of quality of learning experience, mental health, finances, interaction, and mobility. The current study found the pedagogical, logistical, socioeconomic, technological, and psychosocial online learning challenges that students experience within the context of the COVID-19 pandemic. Overall findings indicate that the extent of challenges and strategies varied from one student to another.
**Bernardo 2020 (Research article)**	**Coronavirus Pandemic Anxiety Scale (CPAS-11): Development and initial validation**	To develop an 11-item Coronavirus Pandemic Anxiety Scale (CPAS-11) to measure symptoms of anxiety related to the COVID-19 pandemic to help identify individuals who might need mental health services.	Brings up pandemic anxiety, general anxiety, depression, emotional, social, and psychological wellbeing, positive and negative affect, emotions, and fear of COVID-19.	Online survey to collect primary data using scales such as: Coronavirus Pandemic Anxiety Scale (CPAS-11), Patient Health Questionnare - 9 items (PHQ-9), Mental Health Continuum-Short Form - 14 items (MHC-SF), Positive and Negative Affect Schedule (PANAS), and Fear of COVID-19 Scale (FCV-19S).	Adult population (N=925) in general community settings in different regions across the Philippines.	A two-factor structure was identified and confirmed, corresponding to somatic and non-somatic symptoms of anxiety related to the COVID-19 pandemic. The total scale and subscales showed good internal consistency, convergent, and discriminant validity. Results suggest that the CPAS-11 is a promising tool for screening severity and frequency of COVID-related anxiety and for identifying individuals who might need mental health services. For anxiety (GAD-7 ≥ 15), 13.84% of our participants have severe anxiety, for depression, 21.73% have moderately severe depression (PHQ-9 = 15 to 19), and 9.08% have severe depression (PHQ-9 ≥ 20), and most were still under quarantine.

**Bernardo and Mendoza 2021 (Research article)**	**Measuring hope during the COVID-19 outbreak in the Philippines: Development and validation of the State Locus-of-Hope Scale short form in Filipino**	To develop State Locus-of-Hope Scale to assess hope-related thinking concerning ongoing events and experience during the COVID-19 outbreak in the Philippines in March 2020.	Focused on distress, anxiety, hope and wellbeing.	Survey to collect primary data using the State Locus-of-Hope Scale-Short Form in Filipino, the Mental Health Continuum Short form.	Urban population (N=3182) in a community setting in Metro Manila, Luzon, the Philippines.	Results suggest during the COVID-19 outbreak in Metro Manila, changes in people´s social resources during community quarantine may impact their hope thinking and psychological well-being. Understanding people´s state of hope help towards understanding psychological mechanisms that could buffer the effects of similar pandemics and similar social crises.
**Buenaventura et al 2020 (Opinion piece)**	**COVID-19 and mental health of older adults in the Philippines: A perspective from a developing country**	To discuss the impact of COVID-19 on the mental health of olderFilipinos, who are more vulnerable to the effects of COVID-19, and describe ways that healthcare workers can help alleviate the negative impact on their mental health to the extent possible within the limited resources available in the Philippines.	Notes that community quarantine poses symptoms associated with late-life anxiety and depression. Talks about unmet spiritual needs, poor social wellbeing, decline in physical wellbeing, dying alone, limited access to healthcare services, and health system capacity. Recommends Telehealth, Response Teams that may help manage the care of our sick older patients adequately, and measures to strengthen the national healthcare infrastructure across the country.	NA	Older adult population (N=NA) in general community settings in the Philippines.	Older Filipinos are disproportionately affected by COVID-19. While they constitute less than 8% of the total population, they comprise one-third of all cases and more than half of all deaths related to COVID-19. This underscores what is widely known that older individuals are at a higher risk for COVID-19, with greater morbidity and mortality for this disease. Older Filipinos not only suffer physically but also emotionally, spiritually, and socially. In this unprecedented crisis that developing countries such as the Philippines are not sufficiently equipped to manage, collaborative efforts of the public and private sectors in conjunction with external aids from developed countries and the World Health Organization may help manage the care of our sick older patients adequately.

**Chua et al 2020 (Opinion piece)**	**Changing the Landscape of Medical Oncology Training at the National University Hospital in the Philippines during the Coronavirus Disease 2019 (COVID-19) Pandemic**	To discuss the challenges faced by the Division of Medical Oncology at the national university hospital of the Philippines in terms of subspecialty training and how it has adapted to these difficult predicaments.	Included anxiety, fear, uncertainty, isolation, and burnout, emotional and psychological well-being, among faculty and fellows. Included challenges and adaptations done for the medical oncology training at the national university hospital and a combination of effective traditional and innovative teaching-learning strategies.	NA	Health professionals population (NA) at urban, healthcare/ clinical setting: Division of Medical Oncology, Department of Medicine, University of the Philippines - Philippine General Hospital, Taft Avenue, Manila, Philippines.	Discusses the impact of COVID-19 pandemic on the training of medical oncologists in the Philippines. Identified challenges and adaptations done for the medical oncology training at the national university hospital in the Philippines. Discussed the changing landscape of training, including the combination of traditional and innovative teaching-learning strategies.
**Cleofas 2021 (Research article)**	**Self-care practices and online student engagement during COVID-19 in the Philippines: A mixed methods study**	To determine the relationship between self-care practices and online student engagement, and describe the barriers in practising self-care among undergraduate students during the pandemic.	Focused on stress, disrupted psychological needs, self-care domains, including physical care, mindful awareness, supporting relationships, self-compassion and purpose, supportive structure.	Online survey and interview to collect primary data using the Mindful Self-Care Scale (MSCS) and Online Student Engagement Scale (OSE).	Educator/learner population (N=202): undergraduate students, in an educational setting: De La Salle University, Luzon, in the Philippines	Despite the presence of barriers related to quarantine and online classes, students are able to practise self-care. Older, female students from poorer households and with poor Internet connectivity had significantly lower levels of self-care practices. While self-care has the potential to improve online student engagement, the time-consuming and psychologically exhaustive nature of online classes can hinder students from engaging in meaningful and satisfactory self-care practices. This convergent mixed methods study provides novel empirical evidence on the nuances of students´ engagement in self-care and schoolwork, which can have both a symbiotic and detrimental relationship with each other.

**Cordero 2021 (Letter)**	**Saving our healthcare workers again during the COVID-19 pandemic in the philippines**	To address ways to support HCWs in the Philippines, to highlight that in HCWs are considered as one of the greatest resources against COVID-19, and to propose a "for heroes-welfare" program, for government, school, and church institutions to work hand-in-hand to promote HCWs welfare and support their needs	Brings up depression, anxiety, having inadequate support, experiencing occupational stress, decreased productivity, lack of workplace preparedness, financial concerns associated with changes in in-come and daily living, fear of transmission, and burnout/fatigue. Looks to improve conditions of HCWs, through public appreciation campaigns, education campaigns, and a heroes¬welfare program proposal.	NA	Health professionals population (N=NA) in healthcare/clinical setting in the Philippines.	Healthcare capacity remains a serious concern as of this writing since the number of COVID-19 cases continues to increase due to the more infectious Delta coronavirus variant. If these HCWs lose their patience and run out of hope because of the lack of support from our institutions, then it will be difficult to win this battle against the pandemic. Caring for our HCWs is everybody´s task and it is important for health systems and healthcare organizations to create and ensure programs that will cater to the needs of these unsung heroes.
**Cordero Jr 2022 (Research article)**	**Down but never out! Narratives on mental health challenges of selected college students during the COVID-19 pandemic in the philippines: God, self, anxiety, and depression**	To determine and explore the various themes that play a significant role in the development of this issue by an in-depth study of selected reflection papers submitted in class.	Focused on anxiety, depression, spiritual support, self-awareness, and self-acceptance in mental health.	Document review to collect secondary data (using study student reflection papers).	Educator/learner population: college students (N =6) in educational setting in the Philippines.	College student reflections expressed great effect on their mental health. Themes noted in this study included anxiety and depression as serious effects of the pandemic, and God/Higher Being as the first and/or last source of support, and, the essentiality of self-awareness and self-acceptance in improving mental health.
**Corpuz 2020 (Letter)**	**COVID-19 and mental health**	To discuss findings of the moral responsibility of professionals in helping those who are experiencing mental health problems in the context of the pandemic.	Discussed Telepsychology/ telemedicine; large-scale support system and intervention hotlines to cater to the needs of people who are experiencing anxiety, psychological stress, and posttraumatic stress disorder (PTSD). Notes Project HopeLine, a COVID-19 suicide prevention and crisis helpline) in the Philippines, which saw 200% increase in calls in April 2020.	NA	The Philippines.	The COVID-19 pandemic has had significant social and psychological effects. Medical experts, such as psychiatrists, psychologists, therapists, and counselors, have a moral responsibility to reduce the effects of a mental health crisis. Medical experts, nurses, educators, priests, chaplains, and social workers can also aid in the effort to break the stigma and misconceptions about mental health. Research studies on mental health, suicide prevention, and the current pandemic are needed. Indeed, we need to act collectively to fight the impact of the COVID-19 pandemic.

**Corpuz 2021 (Letter)**	**COVID-19 and mental health issues in the Philippines**	To emphasize that to help those who are experiencing mental health problems, institutions should develop a large-scale support system and intervention hotlines that will cater the needs of people who are experiencing anxiety, psychological stress, and posttraumatic stress disorders.	Discussed stigma, stress, social isolation, anxiety, fear of contagion, emotional and social functioning, posttraumatic stress disorder, suicide, paranoia, effects of lockdown, quarantine, unemployment, and poverty. Brought up interventions such as the 1) the COVID HOPELINE, a phone counseling service - by local church-team composed of mental health experts, priests, and counselors who provide medical, psychological, and moral support for those who are experiencing the psychological impact of COVID-19; 2) educational institutional provision of free mental health counseling services to students, frontline health providers, and those infected with COVID-19; 3) Proposed research studies on mental health, suicide prevention, and the impact of COVID-19 pandemic.	NA	Adult population (N=NA) in general community settings in the Philippines.	The COVID-19 pandemic has had a significant social and psychological effects. Medical experts such as psychiatrists, psychologists, therapists, and counselors trained can include immediate interventions and programs that will reduce the effects of mental health crisis in the Philippines. The author also suggests that medical experts, nurses, educators, priests, chaplains, and social health workers can also help in our collective effort to help break the stigma and misconceptions about the mental health crisis in the Philippines. Research studies on mental health, suicide prevention, and the impact of COVID-19 pandemic are needed to develop preventive measures and interventions to respond to the needs of people who are experiencing mental health concerns.

**Cueto and Agaton 2021 (Research article)**	**Pandemic and typhoon: Positive impacts of a double disaster on mental health of female students in the Philippines**	This study explored the impacts of a double disaster on the mental health of students in the Philippines and how they cope up with these emergencies.	Focuses on anxiety, stress, coping strategies and learning during and following double disasters (typhoon and pandemic).	Online survey to collect primary data using own qualitative questions.	Educator/learner population: (N=6): tertiary school students, in educational settings across different areas of the Philippines (Cagayan, Isabela, Manila, and Oriental Mindoro in Luzon)	Narrative analysis helped find that double disaster affects the total well-being of both individuals and communities as it complicates the challenges brought by the pandemic with natural calamities. Common mental health impacts of the double disaster among the participants were stress and anxiety. Students are particularly vulnerable to mental health problems. The occurrence of natural disasters during the lockdowns from pandemic brought stress to students in adjusting to distance education, completing academic requirements, and accessing technology for online learning. Natural disasters and pandemics act as psychological stressors that trigger suicidal tendencies and thoughts of self-harm. Lack of face-to-face interaction puts stress on the part of students.This was aggravated by the recent typhoons and floods that resulted in a temporary relocation to evacuation centers, loss of learning materials (and properties), and lack of electricity. Financial problems added stress.

**Datu and Fincham 2022 (Research article)**	**The relational and mental health payoffs of staying gritty during the COVID-19 pandemic: A cross-cultural study in the Philippines and the United States**	This study examined how the triarchic model of grit (i.e. perseverance of effort, consistency of interests, and adaptability to situations) is related to cultivation of genuine happiness, loneliness, and COVID-19 anxiety in American and Filipino undergraduate students. It also explored whether grit had indirect effects on such social and well-being outcomes via relatedness needs satisfaction and meaning in life.	Focused on grit, COVID-19-related anxiety, cultivation of genuine happiness, meaning in life, loneliness, relatedness needs and satisfaction.	Online survey to collect primary data using the 10-item triarchic model of grit scale, the 5-item Coronavirus Anxiety scale, 4-item Caring for Bliss Scale, the Meaning in Life Questionnaire, the 8-item UCLA loneliness scale, the 8-items in Relatedness subscale of the Basic Psychological Needs Satisfaction-General scale.	Educators/learners (Ntotal=1189, NPhilippines=546) in educational settings across Manila and Laguna cities, Luzon in the Philippines, and Florida, in the southeast part of the United States of America.	Results of structural equation modeling demonstrated that whereas all grit dimensions were linked to increased relatedness needs satisfaction and meaning in life in the United States, only consistency and adaptability were associated with such constructs in the Philippines. Meaning in life was related to increased cultivation of happiness and reduced loneliness in both societies. Relatedness needs satisfaction was associated with higher happiness as well as decreased COVID-19 anxiety and loneliness in the United States and the Philippines. Evidence supported indirect effects of selected grit´s dimensions on mental health outcomes via relatedness needs satisfaction and meaning in life in both settings.
**Datu et al 2021 (Research article)**	**Are gritty students academically engaged in math and science?**	To examine the cross-cultural differences on triarchic model of grit (TMG) dimensions (i.e., perseverance of effort, consistency of interests, and adaptability to situations) and the associations of grit with academic engagement in Math and Science among secondary school students in Hong Kong, Philippines, and mainland China.	Addressed adaptability, perseverance grit, perseverance, and consistency.	Survey to collect primary data usinf the Triarchic Model of Grit Scale (TMG), Math and Science Engagement Scales 34-item (shortened) and the 5-item conscientiousness scale.	Educators/learners: college students (N=1386, the Philippines) in educational settings across Manila, the Philippines, Hong Kong, and Kunming City, China.	This study provides a preliminary evidence on the cross-cultural variations on grit in selected non-Western settings and the link of these grit´s dimensions to overall academic engagement in Math and Science in Hong Kong, the Philippines, and mainland China. The findings point to the cross-cultural differences in all dimensions of grit with students in the Philippines demonstrating the highest scores on perseverance and adaptability. It is likely that both dimensions of grit may bemore salient in Filipino sample as these students who were drawn from public schools primarily catering for low-income students, tend to experience more adversities associated with socioeconomic challenges and natural calamities.

**Datu et al 2022 (Research article)**	**Psychometric validity and measurement invariance of the caring for bliss scale in the Philippines and the United States**	To explore the Caring for Bliss scale- cross-national invariance among college students in the Philippines and the United States.	Focuses on bliss, wellbeing, depression, anxiety, and stress.	Online survey to collect primary data using the 4-item caring for bliss scale, the 5-item WHO wellbeing scale, the 21-item Depression, Anxiety, and Stress scale (DASS-21).	Educators/learners (Ntotal=1189, NPhilippines=546) in educational settings across the Philippines and the United States of America.	This research contributes to extant assessment literature through offering evidence about the structural validity and construct validity of the Caring for Bliss scale. Scores from the unidimensional model of the caring for bliss construct were valid and reliable among undergraduate students in the United States and the Philippines. Samples indicat(ed) that this construct had similar meanings in these contexts. Findings on the cross-cultural invariance of the Caring for Bliss scale can serve as a stimulus for effectively assessing pursuit of happiness in diverse cultural societies.
**De Los Santos et al 2021 (Research article)**	**Fear of COVID-19, poor quality of sleep, irritability, and intention to quit school among nursing students: A cross-sectional study**	To assess the fear of COVID-19 among prelicensure nurses and its impact on their well-being and intention to leave the nursing education.	About fear of COVID-19, sleep quality, irritability, and the intention to leave nursing education.	Online survey to collect primary data using the Fear of COVID-19 scale (FCV-19S), a single-item from a sleep quality scale, the Brief Irritability test, and a single-item question regarding intent to leave nursing school.	Health professionals: nursing students (N=261) in a healthcare/clinical setting in the Central part, Visayas, in the Philippines.	This study revealed that the fear of COVID-19 is common among nursing students but is more evident in the first year of the program. Although students' fear is correlated to their willingness and preparedness to care for patients with COVID-19, the willingness factor predicts their fear of the virus. We also conclude that the students fear of COVID-19 predicts their psychological disposition of increased irritability, poor sleep quality, and intention to quit nursing school. The first-year nursing students are found to be the most fearful among the group.The students' fear of COVID-19 is associated with their high irritability, poor sleep quality, and desire to quit nursing school.

**Del Castillo et al 2020 (Letter)**	**Correspondence churchinaction: The role of religious interventions in times of COVID-19**	The authors have called for the need to establish psychological support structures that cater to people´s mental health in the time of the coronavirus disease 2019 pandemic. To be more holistic, we extend this call to include people´s spiritual well-being as well. We highlight the initiatives of the Philippines´ religious sector.	Talks about the importance of spiritual wellbeing, spirituality and religion, and interventions such as online-based Church masses, community prayers, spiritual collections.	NA	Religious population (N=NA) in the Philippines.	Reports some of the interventions made by the Roman Catholic Church that have led to the social media hashtag, #ChurchInAction. These religious and spiritual interventions showcase the efforts of the Philippine Church and play an important role in providing assistance in time of public health crisis. They guide people to find meaning and acceptance in the midst of suffering while engaging in the deepest questions of life. They provide counsel, refuge, hope and rejuvenation for those who need it. The COVID-19 crisis calls upon all the members of the global village to understand, learn lessons, reflect and reprioritize all aspects of our lives, the physical, the mental and the spiritual.
**Dy 2021 (Research article)**	**Students' Perceptions and Anxieties towards e-Assessment: Implications for Online Classroom Delivery**	To determine if the students´ anxieties and perceptions have a significant correlation with one another. As well as identifying the challenges that students and teachers face with online classroom delivery and how these affect their perceptions and anxiety levels.	Focuses on students´perceptions and anxieties related to electronic assessments (e-assessments) as a result of the lockdown and other measures during the pandemic.	Online survey (cross-sectional) to collect primary data using the 18-item Perceived Threat of Tests and 24-item Cognitive Test Anxiety (CTAS-2).	Educator/learner population (N=99, including 65 students, 34 teachers) in an educational setting (students in the Science, Technology, Engineering, and Mathematics (STEM) course and teachers in 8 public and private schools) in the Philippines.	This study reported moderate perceived threat category (45.50), and moderate cognitive anxiety (CTAS-2 = 58.86). The teacher-respondents remarked that some of the challenges that they have faced were the lack of a strong internet connection, lack of resources, unfamiliarity with new technology, difficulty assessing students online, lack of training, and conformity to the Data Piracy Act in preparing modules. They have also stated that the students were facing the same problems as well. Students perceive e-assessment as something stressful due to the limitations of the gadgets and technology per household. The subject content is hard-enough for them to learn as is and now they have to deal with technological barriers that hinder learning.

**Edara et al 2021 (Research article)**	**Religiosity and contentment among teachers in the Philippines during COVID-19 pandemic: Mediating effects of resilience, optimism, and well-being**	To explore the relationship between religiosity and contentment among teachers in the Philippines during COVID-19, as well as the mediating roles of resilience, optimism, and well-being.	Addressed stress, burnout, anxiety, uncertainty, as well as resilience, religiosity, optimism, well-being, and contentment.	Online survey to collect primary data using the 20-items interreligious Centrality og Religiosity Scale (CRSi-20), the Brief Resilience Scale (BRS), the Life Orientation Test-Revised (LOT-R), the Teacher Subjective Well-being Questionnaire (TSWQ), and a self-made two item contentment scale.	Educator/learner population: teachers (N=296) in an educational setting in the Philippines.	Results indicated that religiosity has both direct and indirect effects on contentment. Particularly, optimism and well-being had significant mediating effects on contentment. The structural path model suggested a significant path in the direction of religiosity, optimism, well-being, contentment. Religious belief system of the participants was a primary source and the cognitive nature of optimism as an intermediary element might have created a desirable effect on both well-being and contentment of the teachers during the COVID-19 pandemic.
**Egcas et al 2021 (Research article)**	**After over a Year of Pandemic: Mental Well-being and Life Satisfaction of Filipino College Students**	To assess the subjective mental well-being and satisfaction with life of Filipino college students.	Foucsed on poor mental health, fear, lockdown fatigue, loneliness, depression, mental wellbeing, and satisfaction with life.	Online survey to collect primary data using the Short Warwick-Edinburgh Mental Wellbeing Scale, and the Satisfaction with Life Scale.	Educator/learner population: college students (N=1141) in an educational setting in the Western Visayas region of the Philippines.	This study suggests that the rather long-term presence of the COVID-19 pandemic has likely affected college students´mental well-being and psychological health resulting in a looming mental health crisis. Also, students´ mental well-being is a valuable resource that contributes to their life satisfaction. This research found that younger students with lower general self-reported health status tend to have poor subjective mental well-being and life satisfaction.

**Eguia and Capio 2022 (Research article)**	**Teletherapy for children with developmental disorders during the COVID-19 pandemic in the philippines: A mixed-methods evaluation from the perspectives of parents and therapists**	To evaluate teletherapy delivery for children with developmental disorders in the Philippines during the COVID-19 pandemic, from the perspectives of therapists and parents.	About fatigue, exhaustion, service provision, including teletherapy for children with developmental disorders in the Philippines.	Survey and interviews, specifically noting mixed methods, to collect primary data using new (non-standardized) scale.	Health professionals and parents population (N=149) in community setting in the Philippines.	This evaluation showed that stakeholders viewed teletherapy as a satisfactory service model. It appears that a heightened focus on family-centred care was a crucial enabler in utilizing telehealth as a response to the therapy needs of children with developmental disorders during lockdown. Tele-therapy met the needs of children with neurodevelopmental disorders in the Philippines during the lockdown. The interactions of therapists with family members in their natural home environments allowed a heightened focus on family-centred care. Changes in work patterns, as a consequence of the lock-down and working from home, led to increased time and energy requirements that had a negative impact on the well-being of therapists and parents.

**Ferrer et al 2021 (Opinion piece)**	**Experiences, challenges and looking to the future in a clinical tuberculosis cohort in the time of COVID-19 in the Philippines**	To present the current experiences, challenges and obstacles of our team during this period as we attempt to fulfill our roles and responsibilities in Metro Manila, Cebu and Negros Occidental in the Philippines - cohort study of Filipino tuberculosis patients is currently undergoing data collection amidst the corona virus disease 2019 pandemic.	Discussed stigma, anxiety, and fear, as well as access to healthcare during the pandemic.	NA	Health professionals population (N=NA) in healthcare/clinical setting in Metro Manila, Cebu and Negros Occidental in the Philippines.	There were operational challenges of running TB Clinical cohort during COVID-19. The pandemic affected participant´s mental health; there was no policy for regular SARS-CoV-2 screening among the TB afflicted or suspected. Some reported that they were fearful of leaving their homes because they felt more at risk from COVID-19 than from their TB, while others shared their frustration with transport restrictions affecting their access to clinic services. Others reported increased stigma from being visited by HCWs in PPE when delivering drugs. Our experiences demonstrate significant logistical and social challenges, even in a middle-income setting with high mobile penetrance. The underlying themes were similar, covering the supply chain, mobility, communication, physical and mental health and disruption of health services due to reallocation of staff. While we maximized the use of mobile devices, logistical challenges remained.

**Généreux et al 2021 (Research article)**	**The evolution in anxiety and depression with the progression of the pandemic in adult populations from eight countries and four continents**	This study examined the evolution of psychosocial impacts with the progression of the pandemic in adult populations from different countries and continents, and identified, among awide range of individual and country-level factors, which ones are contributing to this evolving psychological response.	Focuses on generalized anxiety and major depressive symptoms.	Online survey to collect primary data using General Anxiety Disorder-7 (GAD-7) and Patient Health Questionnaire (PHQ-9).	Adult population (N=17833, of which in the Philippines=1041 in phase 1, N=1003 in phase 2), in general community settings across multiple countries: the Philippines, Belgium, Canada, England, Hong Kong, New Zealand, Switzerland, and the United States.	Probable generalized anxiety disorder (GAD) and major depressive episode (MDE) were assessed. The independent role of potential factors was examined using multilevel logistic regression. Probable GAD or MDE was indicated by 30.1% and 32.5% of the respondents during phases 1 and 2,respectively (a 7.9% increase over time), with an important variation according to countries/regions (range from 22.3% in Switzerland to 38.8% in the Philippines). This proportion exceeded 50% among young adults (18-24 years old) in all countries except for Switzerland. Beyond young age,several factors negatively influenced mental health in times of pandemic; important factors were found, including weak sense of coherence (adjusted odds ratio aOR = 3.89), false beliefs (aOR = 2.33), and self-isolation/quarantine (aOR = 2.01). The length of the lockdown enforced in the Philippines, spanning nearly six months since the start of the pandemic, is reflected in the results of the study as this country has one of the highest levels of GAD and MDE observed.

**Guillasper et al 2021 (Research article)**	**Protective role of resilience on COVID-19 impact on the quality of life of nursing students in the Philippines**	This study examined the influence of resilience on the COVID-19 impact on QoL among nursing students.	Focuses on stress, anxiety, depression, COVID-19, mental health, quality of life, resilience among nursing students.	Online survey (cross-sectional design) to collect primary data using the adaptations of Brief Resilience Scale (BRS) and COVID-19 Impact on Quality of Life (COV19-QoL) scale.	Health professionals population: nursing students, (N=345) in healthcare/clinical setting: governmentfunded nursing school in Central Luzon in the Philippines.	The COVID-19 outbreak has affected the QoL of nursing students (composite score in the COV19-QoL was 3.35 (SD = .80)). Resilience has a protective influence on the impact on QoL concerning main areas of mental health in the context of the COVID-19 pandemic (composite score in the BRS was 3.04 (SD = .51)). Female nursing students and those in nearby presence of COVID-19 case or unsure of a COVID-19 case near their residence were more vulnerable to the impact of COVID-19 on their QoL. Resilience was a vital psychological factor and personal resource that makes nursing students less susceptible to the negative impact of the COVID-19 outbreak and helps reduce the adverse impact of the pandemic on the QoL of nursing students.
**Hechanova et al 2021 (Research article)**	**Online resilience support groups during the COVID-19 pandemic: the Philippine experience**	To examine the outcomes of an online resilience support group during the COVID-19 pandemic in the Philippines, specifically by describing the extent to which the program improved adaptive coping, non-reactivity, resilience and well-being and decreased stress, depression and anxiety symptoms of participants.	Focused on coping, non-reactivity, depression, anxiety, stress, well-being, and resilience. Examined the Katatagan, an asynchronous online support program.	Online survey to collect primary data using the Brief-COPE Scale, 7-item non-reactivity subscale of Baer´s Five Factor Mindfulness Questionnaire, the Depression, Anxiety, and Stress scale -21 items, the WHO-5 Well-being Index, and the 6-item Brief Resilience Scale (BRS) .	Adult and general population (N=53) in various community settings across the National Capital Region, Luzon and other areas in the Philippines.	This shows evidence of the feasibility of an asynchronous peer support program (Katatagan) that offers participants the opportunity to provide and draw support from others with similar experiences. There is evidence that especially in collectivist cultures, social support cultivates psychological resilience post disasters. Survivors who receive emotional and instrumental support report less problematic mental health outcomes. Results revealed significant improvements in adaptive coping particularly seeking emotional and instrumental support, active coping, and religious coping. The results also showed significant improvements in nonreactivity, psychological well-being and resilience and decrease in depression symptoms.

**Izumi and Shaw 2022 (Research article)**	**A multi-country comparative analysis of the impact of COVID-19 and natural hazards in India, Japan, the Philippines, and USA**	This study investigated the impact of COVID-19 on disaster response and recovery from various types of hazards, with regard to pre-paredness, evacuation, volunteering, early recovery, awareness and knowledge of different types of hazards, and preparedness capacity development, including Typhoon Rolly in the Philippines.	About trauma, stress, and the and impact covid-19 of natural hazards.	Online survey to collect primary data without specified scale.	Population affected by disasters (Ntotal=1049, NPhilippines=268) in community settings across the Philippines: Albay, Camarines, Sorsogon, Catanduanes, plus areas in India, Japan, and the United State of America.	COVID-19 has greatly influenced people´s decision to evacuate, regardless of their nationality or the type of hazard. In each country, 10-20% of respondents stated that they did not evacuate because they were worried about COVID-19. Mental health support must be taken into consideration during COVID-19 recovery. The long-term impacts of COVID-19 in the Philippines included: 1) Reduced household income; 2) impact on individual mental health/experienced stress, and 3) reduced social ties.
**Javed et al 2021 (Opinion piece)**	**Reducing the stigma of mental health disorders with a focus on low- and middle-income countries**	To address various aspects of stigma towards mental health with a focus on LMICs and assess measures to increase help-seeking and access to and uptake of mental health services.	Discusses stigma and legislation, health literacy/education/awareness, and integration of mental health into primary healthcare.	NA	NA	Authors note that the Philippines has the 3rd highest rate of mental health problems in the Western Pacific Region, and that around 6 million Filipinos are estimated to live with depression and/or anxiety. Traditional healers and communal unity based on faith is preferred to resolve mental health crisis in many LMICs, e.g., the Philippines. They note specific mental health anti-stigma programs, including from the Philippines. Some of these are: support groups at a hospital to educate patients and caregivers on specific mental illnesses through psychiatrists; regular radio program to expand the reach of the program; call center to provide information from a directory of psychiatrists; models of healthcare financing for mental illness; public awareness prompting that mental illness is curable and manageable, and help relatives and friends of mentally ill patients understanding and coping with mental illnesses better.

**Kahambing 2021 (Letter)**	**Mental health issues of massage therapists who practice sex work during COVID-19 in the Philippines**	To discuss mental health issues of massage therapists who practice sex work during the pandemic in Tacloban City, Philippines.	Brings up stigma, anxiety, stress, finances and fear of the virus, stressful working environment, and coping mechanisms.	Interview to collect primary data using qualitative methods.	Sex worker population: women (N=Not provided) in community setting in Tacloban City in the Philippines.	Mental health considerations vary among massage therapists, even though having a good and empathetic support group and confidence in one´s belief are potential factors for recovery. The burdens of informants remain disproportionate and differ within work (e.g. instances of implicit body or age shaming as most customers prefer skinny and younger ones) or outside work (e.g. family matters, number of children, partners, etc.). There is the threat of having a client exposed to the virus. The mental health consideration of massage therapists who practice sex work need to be coupled with empathy. The root cause of doing sex work within the massage clinics remains to be the anxiety of getting by due to low socioeconomic status.
**Kahambing 2021 (Letter)**	**Child mental health ethics and positivity: The case of modified community pantries during COVID-19 in the Philippines**	To discuss how modified community pantries are interventions that enable public health practice to reach children.	Focuses on child mental health and modified community pantries as an intervention.	NA	Child population (N=NA) in general community settings in the Philippines.	This letter emphasizes that there is positivity exhibited by the modification of community pantries for children´s mental health. We can modify our existing mental health interventions to be focused on children and the modifications need the collective efforts of the community and society. Emphasizes strong social cohesion and that modified community pantries show promising mental health interventions that rightly address the future of children.

**Labrague 2021 (Research article)**	**Pandemic fatigue and clinical nurses´ mental health, sleep quality and job contentment during the COVID-19 pandemic: The mediating role of resilience**	This study examined the influence of pandemic fatigue on clinical nurses´ mental health, sleep quality and job contentment, with resilience as a mediator.	Focused on pandemic fatigue, resilience, job contentment, and sleep quality, and also mentioned sufficient staffing and vaccine access.	Online survey to collect primary data using the Pandemic Fatigue Questionnaire, the Brief Resilience Scale (BRS), Job Contentment Scale, and the Sleep Quality Scale.	Health professional population: nurses (N=255) in healthcare/clinical setting in the Central Region, Visayas, in the Philippines.	Clinical nurses who received a COVID-19 vaccine and those who perceived sufficient staffing in their units reported lower levels of pandemic fatigue. Resilience reduces the effects of pandemic fatigue on clinical nurses´ mental health, sleep quality and job contentment. Overall, clinical nurses reported moderate levels of fatigue, with nurses who received a COVID-19 vaccine and those who perceived adequate staffing reporting significantly lower levels of pandemic-related fatigue. Further, resilience was seen to reduce the negative effects of pandemic fatigue on clinical nurses´ mental health, sleep quality and job contentment.
**Labrague 2021 (Research article)**	**Social and emotional loneliness among college students during the COVID-19 pandemic: The predictive role of coping behaviors, social support, and personal resilience**	To determine the influence of coping behaviors, resilience, and social support on students' emotional and social loneliness during the COVID-19 pandemic.	About loneliness, resilience, coping behavior, perceived social support, and personal resilience.	Survey to collect primary data using the 6-item Loneliness scale, 4-item Brief Resilience scale (BRS), modified Coping Behavior Questionnaire, and the 6-item Perceived Social Support questionnaire.	Educators/learners: college students (N=303) in an educational setting in the Central region, Visayas, in the Philippines.	This study suggests that disease control measures (e.g., mandatory lockdown and social distancing) to contain the virus have increased the levels of loneliness, particularly in younger and female students. Students who had higher levels of personal resilience and coping behaviors, and those who perceive greater social support, reported alower level of loneliness.

**Labrague 2022 (Research article)**	**Specific coping styles and its relationship with psychological distress, anxiety, mental health, and psychological well-being among student nurses during the second wave of the COVID-19 pandemic**	To identify specific coping skills that contribute to relieving anxiety and stress while supporting student nurses' mental health and psychological well-being.	About coping behavior, anxiety, wellbeing, mental health, stress, and interventions such as mindfulness therapy, cognitive behavioral therapy and body and mind interventions (e.g., bio feedback, guided imaginary, relaxation techniques and breathing exercise).	Survey to collect primary data using the Coping Behavior Questionnaire (COPE), the 4-item perceived stress scale (PSS), the COVID-19 Anxiety Scale, the Pscyhological Well-Being Scale, and a 1-item of students perception on mental health.	Health professionals population (N=261) in healthcare/clinical settings in the Central Philippines, Eastern Visayas (Region 8).	Nursing students surveyed reported moderate stress during the current pandemic. Overall, 57.9% of the student nurses identified as having dysfunctional anxiety levels. Despite the moderate stress levels that the student nurses experienced during the second wave of the pandemic, their overall health and well-being were good to excellent. Students reported employing both problem-centered (e.g., seeking support, consultation, resilience) and emotion-centered (e.g., use of social networks to obtain information, excessive eating, alcohol use) coping behaviors when dealing with stress and anxiety associated with the current pandemic.
**Labrague and Ballad 2021 (Research article)**	**Lockdown fatigue among college students during the COVID-19 pandemic: Predictive role of personal resilience, coping behaviors, and health**	To examine the levels of lockdown-induced fatigue and its association with personal resilience, coping skills, and health in college students.	Addressed fatigue, decreased motivation, anxiety, fear, stress, resilience, coping behavior, and perceived general health.	Survey to collect primary data using the Lockdown Fatigue Scale, Brief Resilience Scale (BRS), and the Coping Behaviors Questionnaire.	Educator/learner population: college students (N=243) in various educational settings across different colleges and universities in Western Samar. Eastern Visayas, in the Philippines.	Mandatory lockdown or home confinement measures to slow the transmission of COVID-19 may cause considerable levels of fatigue in college students. Female students, as well as those in the lower levels of education, were found to experience more fatigue than male and graduating students. Further, this study provided empirical evidence linking higher personal resilience and coping skills with decreased levels of lockdown-induced fatigue in students.

**Labrague and De Los Santos 2021 (Research article)**	**Resilience as a mediator between compassion fatigue, nurses' work outcomes, and quality of care during the COVID-19 pandemic**	This study aims to examine the mediating role of resilience in the relationship between Compassion Fatigue and frontline nurses' job outcomes (job satisfaction and turnover intention) and care quality.	Addressed compassion fatigue, quality of care, and job satisfaction.	Online survey to collect primary data using 13-item short version of the Compassion Fatigue Scale (CFS), the Brief Resilient Coping Skills and Job Satisfaction Scales.	Health professionals population: nurses (N=270) in healthcare/clinical setting in the Philippines.	This study was the first to report the consequences of compassion fatigue in frontline nurses during the pandemic in terms of job outcome and quality of care. This study found that the pandemic has contributed to compassion fatigue among frontline nurses in the Philippines, which has adversely affected their work outcomes as well as the quality of care provided in their respective units. Psychological resilience was identified as a protective factor against CF, resulting in higher job satisfaction, increased retention, and a higher perception of quality of nursing care.
**Labrague and De Los Santos 2021 (Research article)**	**Prevalence and predictors of coronaphobia among frontline hospital and public health nurses**	To determine the prevalence as well as the predictors of coronaphobia in frontline hospital and public health nurses.	Focused on anxiety, resilience, social support, perceived health, and coronaphobia.	Survey to collect primary data using the Coronavirus Anxiety scale, the Brief Resilience scale (BRS), the Perceived Social Support questionnaire, amd a single-item measure for perceived health.	Health professionals: nurses (N=736) in healthcare/clinical settings in Eastern Visayas (Region 8) in the Philippines.	Coronaphobia is prevalent among frontline Filipino nurses, with more public health nurses experiencing coronaphobia than hospital nurses. Heightened anxiety related to coronavirus was commonly observed in female nurses, those who held contracted job status, and those who were married. As level of personal resilience predicted COVID-19 anxiety in nurses, interventions geared towards enhancing resilience in nurses through evidence-based education and training are essential to strengthen nurses' defences against the emotional, mental, and psychological consequences of the pandemic. Future studies should focus on testing interventions to improve nurses' resilience to effectively bounce back from adversity and effectively cope with stress caused by the coronavirus pandemic.

**Labrague and De Los Santos 2021 (Research article)**	**Fear of COVID-19, psychological distress, work satisfaction and turnover intention among frontline nurses**	To examine the relative influence of fear of COVID-19 on nurses' psychological distress, work satisfaction and intent to leave their organisation and the profession.	About psychological distress, peer and social support, mental and psychosocial health services, and COVID-19 training.	Survey to collect primary data using the Fear of COVID-19 scale, the Job Stress scale, the Job Satisfaction scale, and a two-single item measures of turnover intention.	Health professionals: nurses (N=261) in healthcare/clinical settings across five urban hospitals in the Philippines.	Results suggest that Filipino frontline nurses experience mild-to-moderate levels of fear of COVID-19. Job status and attendance of COVID-19 training were seen to explain the fear of COVID-19 among frontline nurses, with part-time or contracted nurses and those who had not attended such training reporting increased lev-els of fear of COVID-19. Further, higher levels of fear of COVID-19 were associated with increased psychological distress, lower job satisfaction, decreased health perceptions and increased turnover intention.
**Labrague and Santos 2020 (Research article)**	**COVID-19 anxiety among front-line nurses: Predictive role of organisational support, personal resilience and social support**	This study examines the relative influence of personal resilience, social support and organisational support in reducing COVID-19 anxiety in front-line nurses.	Focuses on anxiety, resilience/coping behavior, social support, organizational support, and mental health services.	In-person survey (cross-sectional study) to collect primary data using: Coronavirus Pandemic Anxiety Scale (CPAS-11), Brief Resilient Coping Scale (BRCS), Perceived Social Support Questionnaire (PSSQ), and the Perceived Organizational Support (POS) questionnaire.	Health professionals population: nurses (N=325) in a healthcare/clinical setting, in Region 8 (Eastern Visayas), in the Philippines.	Front-line nurses in the Philippines reported moderate levels of personal resilience and perceived moderate levels of social and organisational support during the COVID-19 pandemic. A total of 123 (37.8%) were found to have dysfunctional levels of anxiety. Nurse characteristics were not associated with COVID-19 anxiety. Increased levels of personal resilience, organisational support and social support in nurses were associated with decreased levels of anxiety related to COVID-19.

**Lee et al 2021 (Research article)**	**Analysis of personal and national factors that influence depression in individuals during the COVID-19 pandemic: a web-based cross-sectional survey**	To analyze the factors that affected depression in the public during the COVID-19 pandemic to provide evidence for COVID-19-related mental health policies and to emphasize the need to prepare for mental health issues related to potential infectious disease outbreaks in the future.	Analyzed depression, the COVID-19 stringency score, and COVID-19 deaths.	Online survey to collect primary data using the Patient Health Questionnaire-9 (PHQ-9), and the Oxford COVID-19 Government Response Tracker, Legatum Prosperity Index.	Adult and general population (Ntotal=2683, NPhilippines=288) in several community settings across the Philippines, China, Democratic Republic of Congo Republic of Korea, Ethiopia, Japan, Paraguay, and Peru.	This article shows a COVID-19 stringency score in the Philippines of 93. Participants in the Philippines (288) made up 10.13% of this sample and showed that a higher than overall median (6) in the Philippines suggesting a higher level of depression compared to other countries. This paper also shows data on social capital, life expectancy, and PPP in each country.
**Leochico et al 2020 (Literature review)**	**Challenges to the Emergence of Telerehabilitation in a Developing Country: A Systematic Review**	To determine the challenges faced by telerehabilitation in the Philippines.	Investigated intervention: Telerehabilitation, a subset telemedicine and an emerging technology using electronic means in remotely conducting evaluation, consultation, therapy, and monitoring to provide rehabilitation care for patients in various locations, including for mental health.	No collection method of primary data. Secondary data collected via databases: PubMed, Scopus, Embase, CochraneLibrary, and HeRDIN.	Health professionals and patient population: Patient and physician population: engaged in telerehabilitation in the Philippines.	There is paucity of data on telerehabilitation in the Philippines. There are human, organizational, and technical challenges to telerehabilitation or telehealth. Identified 18 human, 17 organizational, and 18 technical unique challenges related to telerehabilitation in the Philippines. The most common challenges were slow internet speed (in 10 studies), legal concerns (9), and skepticism (9). Patients sought telemedicine or telerehabilitation for general medical conditions, chronic diseases, mental health issues, orthopedic problems, neurologic conditions, communication disorders, and cardiac conditions.

**Leones et al 2020 (Report)**	**Caring for the carers: Safeguarding oncologists' mental health in the time of COVID-19**	To discuss how the Division of Medical Oncology at Philippine General Hospital employed the following measures to safeguard the mental health of its faculty and fellows during the COVID-19 pandemic: 1) use of psychological support materials; 2) initiation of a psychological intervention programme and 3) establishment of peer support programmes.	Included oncologists´ emotional and cognitive coping mechanisms and how they adapted to the stresses of the pandemic. Highlighted psychological support materials and peer support to address anxiety, stress, etc. while the Cancer Institute remained operational, working on a skeleton workforce, during a time when the hospital was a national COVID-19 referral centre.	Online survey, and online group discussions to collect primary data using standardised questionnaires on anxiety, depression and burnout (specific scale not provided).	Health professionals population (N=12 surveys, N=16 for online group discussions), at urban, healthcare/ clinical setting: Division of Medical Oncology, Department of Medicine, University of the Philippines - Philippine General Hospital, Taft Avenue, Manila, Philippines.	The Division of Medical Oncology at the University of the Philippines-Philippine General Hospital employed the following measures to safeguard the mental health of its faculty and fellows: 1) use of psychological support materials; 2) initiation of a psychological intervention programme and 3) establishment of peer support programmes. Caring for the carers through evidence-based interventions ensures the delivery of quality care to our cancer patients despite the challenges during COVID-19.
**Lim et al 2022 (Protocol)**	**Assessing the effect of the COVID-19 pandemic, shift to online learning, and social media use on the mental health of college students in the Philippines: A mixed-method study protocol**	To assess Filipino college students´ mental health status and to estimate the effect of the COVID-19 pandemic, the shift to online learning, and social media use on mental health.	About depression, anxiety, stress, resilience/coping, and facilitators or stressors that modified mental health status.	Online survey and interview to collect primary data using the Depression, Anxiety, and Stress Scale (DASS-21) and Brief-COPE.	Educator/learner population: Red Cross Youth (RCY) (N=NA) in an educational setting in the Philippines.	This protocol focuses on the COVID-19 pandemic and its accompanying effects affecting the mental wellbeing of college students. The results of this proposed study will tailor the possible coping strategies to meet the specific needs of college students nationwide, thereby promoting psychological resilience.

**Malaluan et al 2022 (Opinion piece)**	**Impact of COVID-19 Pandemic on The Physical and Mental Health of Adolescents in The Philippines**	To discuss the impact of the COVID-19 pandemic on the physical and mental health of adolescents in the Philippines.	About suicide, and the Mental Health Act.	NA	Adolescent population (N=NA) in community setting in the Philippines.	The COVID-19 pandemic threatens Filipino adolescents´physical and mental health,. Available data on the mental health condition of Filipino adolescents infected with COVID-19 are lacking. However, the National Center for Mental Health noted a drastic increase in suicide-related calls (400 calls per month) in 2021 versus thepre-pandemic period (80 calls per month). Filipino adolescents also face multiple threats to mental health, includingnatural disasters, poverty and the risk of child abuse, neglectand exploitation. These conditions put them at higher risk ofmental health disorders when infected with COVID-19. Despite passing the Philippine Mental Health Act (RA 11036), access to mental health care remains limited. Most paediatricians, adolescent medicine specialists, and psychiatrists practicein urban areas in the country. Moreover, payment for mentalhealth consultation remains an out-of-pocket expense for Filipinos.
**Malolos et al 2021 (Opinion piece)**	**Mental health and well-being of children in the Philippine setting during the COVID-19 pandemic**	To highlight the scope of the mental health epidemic in Filipino children during the COVID-19 pandemic.	Discusses effects on children including depression, anxiety, sterss, sleep disruptions, fear, child labor and online child abuse reports. It also discusses general interventions such as the Philippine Red Cross´ COVID-19 hotline and Department of Health nationwide campaigns to address mental health, and emphasizes scarcity of children-centered interventions.	NA	Child population (N=NA) in general community setting in the Philippines.	The already-challenged state of mental well-being of Filipino children has been worsened by the pandemic and the lack of good mental health policies by the government. While there is increasing awareness for mental health, children-centered interventions remain deficient. Approaches must integrate commonly-known mental health effects on children with existing and anticipated Philippine societal issues. Without doing so, it may be expected that as the COVID-19 pandemic is mitigated, a mental health epidemic will replace it.

**Marzo et al 2021 (Research article)**	**Psychological distress during pandemic COVID-19 among adult general population: Result across 13 countries**	To survey the general population across 13 countries (Bangladesh, Egypt, India, Indonesia, Iran, Malaysia, Myanmar, Nepal, Philippines, Sri Lanka, Thailand, Turkey, and Vietnam) to measure the prevalence and severity of psychological distress.	Focused on distress, stress, anxiety, and depression.	Online survey to collect primary data using the COVID-19 Peritraumatic Distress Index.	Adult population (Ntotal=7091, NPhilippines=357) in educational setting (research networks of universities, hospitals, friends and their relatives), across the Philippines, Bangladesh, Egypt, India, Indonesia, Iran, Malaysia, Myanmar, Nepal, Sri Lanka, Thailand, Turkey, and Vietnam.	Vietnam had the highest prevalence of psychological distress followed by Egypt, where Nepal had the least. The prevalence of distress in the Philippines was (185/357), 51.8%, and univariate, (6.55), multivariable (6.10), and log reg (6.58), all showed significant (>0.001) COVID-19 distress. The study findings indicate that psychological distress varies across different countries.
**Mendoza et al 2022 (Research article)**	**The factor structure and measurement invariance of the phq-4 and the prevalence of depression and anxiety in a southeast asian context amid the COVID-19 pandemic**	To examine the psychometric properties of the Patient Health Questionnaire-4 (PHQ-4) as an ultra-brief screener of depression and anxiety in the Philippines during the first few months of the COVID-19 outbreak.	Focused on depression, anxiety, and health promotion materials via survey linked	Online survey to collect primary data using PHQ-4, DASS-S, PHQ-9, and GAD-7.	Adult population (N=4524) in community settings in Southern Luzon, the National Capiital Region (NCR), and Central Luzon in the Philippines.	Findings support the PHQ-4 instrument´s reliability and validity. The two-factor structure of the PHQ-4 held across configural, metric, and scalar invariance tests demonstrating the scales, and robust ability to assess depression and anxiety symptoms across age, gender, and locale. The PHQ-4 is a reliable, valid, and cost-effective measure of depression and anxiety symptoms. In estimating prevalence rates, among those screened by the PHQ-4 cut-off scores for depression (n = 1905, 42.11%) and anxiety (n = 1853, 40.96%), 81.78% and 94.06% were consequently screened by the PHQ-9 and GAD-7, respectively.

**Mostajo et al 2021 (Research article)**	**Discovering the resilience of working women from academic institutions in the Philippines during the COVID-19 pandemic crisis**	To investigate the resilience of Filipino women employed in educational institutions during the COVID-19 pandemic as they tried to balance their roles as wives, mothers, and workers. It specifically explored these research objectives: (1) to know the level of resilience of the Filipino working women; (2) to recognize the challenges experienced by working women from educational institutions in the Philippines during the COVID-19 pandemic and the effects of these challenges to them; and (3) to discover how the Filipino working women in the academe coped with these challenges.	Focused on stress, mental exhaustion, emotional disturbances, and resiliency.	Online survey/questionnaire and interviews to collect primary data using the Modified Personal Resilience Scale (PRS).	Educator/learner population: women in academia (N=326) in various community settings across Luzon, Visayas, and Mindanao in the Philippines.	The findings indicated that the Filipino mothers working in academia have a very high level of resilience manifested by their resiliency characteristics and behaviors, thus having the strong capacity to recover quickly from the difficulties they may have experienced during this pandemic.This claim is substantiated by the results of the PRS, and the various themes conceptualized from the participants´ online responses in the open-ended questions. Themes reflecting working women´s ways of coping: self-efficacy, social relatedness, spiritual connectedness, self-nurturance.
**Narvaez 2022 (Literature review)**	**Benefits and challenges of telepsychiatry services in SouthEast Asian nations during the COVID-19 era: An integrative review**	To examine current evidence on the benefits and challenges of telepsychiatry services of SouthEast Asian nations, namely Brunei, Myanmar, Cambodia, Timor-Leste, Indonesia, Laos, Malaysia, Philippines, Singapore, Thailand and Vietnam.	About grief and telepsychiatry services.	Document review to collect secondary data.	NA	This integrative review presented additional support on the benefits and challenges of telepsychiatry services in SouthEast Asian nations during the COVID-19 pandemic. This integrative review showed little to no evidence of telepsychiatry services within SouthEast Asian nations. The concept or notion of telepsychiatry services (e.g. telemedicine in mental health, telemental services, e-health, e-mental health, digital psychiatry and digital health interventions) within SouthEast Asian nations is exceptionally novel and needs further research in the medical and allied health discipline.

**Nashwan et al 2022 (Research article)**	**Stigma towards health care providers taking care of COVID-19 patients: A multi-country study**	To investigate the prevalence of stigmatization during the COVID-19 pandemic among HCPs in seven different countries using the Stigma COVID-19 Healthcare Providers tool (S19-HCPs).	About stigma (fear of get-ting infected with COVID-19) and stress.	Survey to collect primary data using the Stigma COVID-19 Healthcare Providers (HCPs) scale.	Health professionals; (Ntotal=1726, NPhilippines, 311) Healthcare/clinical settings across the Philippines, Egypt, Iraq, Jordan, Kuwait and Saudi Arabia.	Findings concluded that participants from the seven countries overall, although having perceived high levels of stigmatization, still observe positively by their respective communities and, in their utmost, highly motivated to care for COVID-19 patients. There are still lurking fears of discrimination among HCPs. The levels of fear were higher among the Filipino healthcare providers with the mean of 1446.1. Despite the level of fear, Filipino healthcare providers showed the highest score in their readiness to care for COVID-19 patients. Furthermore, Filipino healthcare providers think that they are perceived well while caring for COVID-19 patients and they also obtained the highest mean score in the willingness to follow precautionary measures (1379.17).
**Nu Htay et al 2022 (Research article)**	**Digital health literacy, online information-seeking behaviour, and satisfaction of COVID-19 information among the university students of East and South-East Asia**	This study aimed to investigate the digital health literacy (DHL) level, information-seeking behaviour, and satisfaction of information on COVID-19 among East and South-East Asia university students.	Focsued on fear and stress.	Online survey to collect primary data using COVID-Health Literacy University Students Questionnaire and the Digital Health Literacy Instrument (DHLI).	Educator/learner population: college students (Ntotal=5302, NPhilippines=2307) in educational settings across the Philippines, China, Hong Kong, Macau and Malaysia.	Revealed that digital health literacy is a key concept to utilize trustworthy online sources and to achieve higher satisfaction with online information sources, which in turn could be beneficial in making appropriate decisions related to their health. In the Philippines, university students reported that fear of getting infected is significantly associated with the utilization of Facebook as the information source (p=0.035). Findings revealed that safeguarding privacy was positively associated with the utilization of trust worthy online resources among Chinese and the Philippines students.

**Ong et al 2021 (Research article)**	**The impact of the coronavirus disease 2019 pandemic on gastroenterologists in Southeast Asia: A mixed-methods study**	To determine the impact of the pandemic on clinical demands including burnout among gastroenterologists within the region, and to identify risk factors for burnout and determine regional stressors.	About stress (pandemic-related stressors and general stressors), burnout, emotional exhaustion, depression, and depersonalization.	Survey, including qualitative data to collect primary data using the 22-item Maslach Burnout Inventory-HumanServices Survey (MBI-HSS).	Health professionals: Gastroenterologists (Ntotal=683, NPhilippines=135) in healthcare/clinical settings across the Philippines, Brunei, Indonesia, Malaysia, Singapore, and Thailand.	This study showed that burnout affects gastroenterelogists. The majority of respondents were male, and this suggests that the speciality has a male predominance in certain Southeast Asian counties. 50.1% of all gastroenterologists who participated in this study reported that they were unaware of or did not have access to support services, and this was associated with an almost two-fold increase in burnout risk. Proportions varied between countries (34.9%-70%) with the Philippines (58.9%) in the middle. In countries with predominant private health-care systems, for example, the Philippines, gastroenterologists reported that they were encountering fewer but sicker patients, in addition to a reduction in income.
**Pagaling et al 2022 (Research article)**	**The practice of teleneurology in the Philippines during the COVID-19 pandemic**	To describe the practice of teleneurology among Filipino neurologists and determine the factors affecting its adoption using the unified theory of acceptance and use of technology (UTAUT) model and its constructs, namely performance expectancy, effort expectancy, social influence, and facilitating conditions.	Foused on teleneurology (part of telemedicine).	Online survey to collect primary data using a 22-item checklist based on literature review and key informant interviews from telemedicine /neurology, and the 17-item UTAUT questionnaire.	Health professionals neurologists (N=147) in a healthcare/clinical setting in the Philippines.	The UTAUT model and the construct of Performance Expectancy (PE) and Facilitating Conditions (FC) provide significant explanatory power on the adoption of teleneurology in a resource-limited setting. The majority of the participants have practiced teleneurology during the pandemic utilizing social media applications via videoconferencing through desktop/laptop. Only half of them were affiliated to an institution with teleneurology and had to learn on their own or through their colleagues.

Palattao et al 2021 (Research article)	**Determining factors contributing to the psychological impact of the COVID-19 Pandemic using machine learning**	This study aims to determine the possible contributing factors to stress, anxiety, depression, and adverse psychological impact on the general population of the Philippines resulting from the pandemic using machine learning approaches.	Focuses on mental health, stress, anxiety, depression, and the psychological impact of COVID-19. Also features machine learning and feature selection.	Online survey to collect primary data using Depression, Anxiety and Stress Scale - 21 Items (DASS-21) and Impact of Event Scale - Revised - 22 items (IES-R).	Educator/learner population: network of alumni (N=2119), in an educational setting: University of the Philippines in Luzon, the Philippines.	Machine learning techniques were used in this research to generate predictive models to help identify possible cases and predictors of mental health conditions among the general population in the Philippines. The results show that longer hours at home, on social media, age, how people rate their own health, pre-existence of a neuropsychiatric condition; wanting information on availability and effectiveness of a medicine or vaccine, being concerned for their family, feeling discriminated; and symptoms of body pain, difficulty breathing, and cough were good predictors of individuals being adversely impacted psychologically by the pandemic and others having elevated levels of stress, anxiety, depression. This research supports machine learning in the field of detection and diagnostics of mental health conditions.
Rabacal et al 2020 (Research article)	**COVID-19 impact on the quality of life of teachers: A cross-sectional study**	To determine the impact of the COVID-19 pandemic on the quality of life primarily related to the mental health of licensed professional teachers in the Philippines.	The COVID-19 Impact on Quality of Life (COV19-QoL) was the primary measure for this study and was utilized to capture the effect of COVID-19 on the main areas covering the QoL in relation to mental health and the six-item Perceived Coronavirus Threat Questionnaire (PCTQ) was used to assess COVID-19 threat-related thoughts and worries.	Online survey (descriptive, cross-sectional research design) to collect primary data using COVID-19 Impact on Quality of Life (COV19-QoL), and the 6-item Perceived Coronavirus Threat Questionnaire (PCTQ).	Educator/learner population: Professionally licensed teachers enrolled in the master's and doctoral program majoring in Educational Management (N=139) in educational setting: at a government-funded college, (authors from Iloilo, Visayas), in the Philippines	This study found that the mean composite score of teachers in the COV19-QoL scale was 2.75 for nearly six months since the Philippines implemented strict community quarantine. This study found no significant difference in the impact of COVID-19 on QoL based on perceived threat and between those with either knowledge of the presence or absence of any COVID-19 cases near their residence. The findings also indicated that the greatest impact of the pandemic among teachers was on their personal safety followed by quality of life in general whereas mental health was the least impacted.

**Rocha et al 2021 (Opinion piece)**	**Typhoons during COVID-19 Pandemic in the Philippines: Impact of Double Crises on Mental Health**	To address the effects of natural disasters such as typhoons on the mental health of Filipinos during the COVID-19 pandemic.	Talks about poor mental health, stress, depression, anxiety, post-traumatic stress disorder (PTSD) resulting from natural disasters and the pandemic. Discusses interventions such as telemedicine, coping strategies, psychological first aid, debriefing, cognitive behavioral therapy, psychosocial support, and community-based interventions), and integrating mental health into primary care.	NA	Disaster-affected populations (N=NA) in various community settings in the Philippines.	The Philippines experienced many natural disasters during the pandemic. Typhoons in 2020 had devastating infrastructural impacts on an already poverty-stricken country, with the addition of a growing COVID-19 pandemic. Loss of basic amenities, homelessness, financial stress, and lack of social distancing in evacuation centers were prevailing factors to mental instability. Among the psychological impacts, depression, anxiety, and PTSD were increasing within a population that already had high mental health problems. International agencies and the government are making efforts to recover, including mental health support services. Examples include Regional office of the Department of Health (DOH) in Bicol training workers in rural and city areas to manage mental health concerns within their communities; government units to increase mental health institutes (e.g., Don Susano Rodriguez Memorial Mental Hospital); local government units with Department of Education conducting therapeutic sessions on psychological well-being and mental support programs; and initiatives to provide psychological first aid training to teachers to address children´s mental health issues.

**Sawicki et al 2022 (Research article)**	**The fear of COVID-19 scale: Its structure and measurement invariance across 48 countries**	To evaluate the psychometric properties of the most popular relevant instrument, the Fear of COVID-19 Scale exploring the measurement invariance and cross-cultural replicability and testing community samples from 48 countries.	About fear of COVID-19, anxiety, and stress.	Online survey to collect primary data using the Fear of COVID-19 Scale (FCV-19S), the 6-item STAI-6, and the 4-item PSS-4.	Adult population (Ntotal=14557, totalPhilippines=188) in community settings across 48 countries in the Philippines, Armenia, Australia, Austria, Bangladesh, Bosnia and Hercegovina, Brazil, Bulgaria, Chile, China, Colombia, Croatia, Czech, Ecuador, Estonia, Ghana, Hungary, India, Indonesia, Iran, Iraq, Israel, Italy, Japan, Kazakhstan, Lativa, Lebanon, Malaysia, Nigeria, Pakistan, Peru, Poland, Portugal, Romania, Russia, Serbia, Slovakia, Slovenia, South Africa, Spain, Thailand, Togo, Turkiye, Ukraine, United Arab Emirates, United Kingdom, Uruguay, and Vietnam.	Examined the scale in 48 countries and found that it is unifactorial. However, also found that the scale is in need of measurement modifications, which currently limit its utility. Factor analyses indicated that the scale assesses two aspects of fear of COVID-19, psychological and physiological, but the issue needs more thorough investigation. Although the scale is suitable for cross-cultural research, it is limited when it comes to examining correlates and predictors of fear of COVID-19. We suggestthat the FCV-19S might be cautiously used in studies focused oncross-cultural comparisons of level of fear of COVID-19. The Fear of COVID-19 (mean=3.84). anxiety (mean=2.78), stress (mean=2.81) correlations for the Philippines.
**Shaikh et al 2021 (Research article)**	**COVID-19 and mental health: a multi-country study- the effects of lockdown on the mental health of young adults**	To assess the mental health of young adults in Egypt, Ghana, India, Pakistan, and the Philippines during the COVID-19 lockdown. It further reviews the coping mechanisms used by the youth of these countries.	Focuses on depression, anxiety, stress, and coping.	Online survey to collect primary data using the Depression, Anxiety, and Stress scale (DASS-21), and the Brief-COPE 28-item scale.	Young adult population (N=526) in various community settings across the Philippines, Egypt, Ghana, India, and Pakistan.	The findings of the study confirm that COVID-19 lockdown has affected the mental health of young adults. In particular, the presence of negative coping strategies used by the youth tends to be an indicator of increased levels of stress, anxiety, and depression and it should be considered when planning interventions within this population.

**Skinner et al 2021 (Research article)**	**Parent-adolescent relationship quality as a moderator of links between COVID-19 disruption and reported changes in mothers, and young adults, adjustment in five countries**	To examine mother and young adult reports of disruptions and adjustment during the COVID-19 pandemic and how prior parent-child relationship qualities assessed during adolescence moderate the relation between personal disruption during the pandemic and adjustment outcomes for young adults and their mothers.	About parenting, anxiety, depression, and supportive parent-child relationships.	Telehpone and online interviews to collect primary data using the Knowledge, Disclosure, Control, and Solicitation Scale, and a developed 19-question measure about COVID-related experiences.	Parent and child population (Ntotal=466, NPhilippines=60) in community settings across Manila, the Philippines, Chiang Mai, Thailand, Durham, North Carolina, United States, and Trollhättan/Vänersborg,Sweden.	This paper reveals that higher levels of reported disruption during the pandemic are related to reported increases in internalizing and externalizing behaviors after the onset of the COVID-19 pandemic for young adults (Mage=20) and their mothers in all five countries (including the Philippines), with the exception of one association in Thailand. For most mothers supportive parenting likewise attenuated the impact of pandemic-related disruptions on internalizing and externalizing behavior. Associations between disruption during the pandemic and young adults and their mothers, reported increases in internalizing and externalizing behaviors were attenuated by higher levels of youth disclosure, more supportive parenting, and lower levels of destructive adolescent-parent conflict prior to the pandemic.
**Sunga et al 2021 (Research article)**	**The 'plantito/plantita' home gardening during the pandemic**	To explore and describe the rise of (plantito/plantita) home gardening among Filipinos during the pandemic. It will explore the conditions and situations surrounding this home gardening during the pandemic based on participant perspective, discovery, motivations, and how it will influence their mental health as well as their community.	Discusses stress and home gardening as an intervention. Brings up positivity, good well-being, and happiness, and relief from stress, anxiety, and boredom.	Online survey to collect primary data using no specified scale.	People who garden (N=104) in the general community settings in the Philippines.	Participants connected being plantito/plantita as something experiential, associated with psychological benefits such as positivity, good well-being, and happiness. The opportunity to try home gardening, considering the community quarantine restrictions provided relief from stress, anxiety, and boredom. Plants were cited as as a sign of hope, aesthetics, urban sustainability, and alternate source of food. The mental health benefits included stress relief from improved mood and sense of purpose and accomplishment.

**Tan-Lim et al 2022 (Research article)**	**Assessing the Impact of a Physician Resiliency and Wellness Program to Physician Burnout Levels in a Pediatric Department of a Tertiary Hospital: A Pilot Study of the I-CARE Program**	To evaluate the effectiveness of the I-CARE program in reducing medical residents´ burnout level.	About burnout, stress and the I-CARE program (physician resiliency program) at the Department of Pediatrics at Philippine General Hospital.	Online survey and interview to collect primary data using the Maslach Burnout Inventory.	Health professionals population: residents (N=118) in a healthcare/clinical setting: Philippine General Hospital, Luzon, in the Philippines.	Showed that 53 out of the 59 respondents (89.83%) from the Department of Pediatrics had high levels of burnout. Although the tool used to measure burnout differed in the 2016 study (ProQOL), it is nevertheless alarming to note such high rates of burnout. The study failed to demonstrate a direct correlation with the implementation of the I-CARE program and a decrease in medical resident burnout or attrition rates, but still yielded some improvement in burnout scores and had positive feedback among the participants as evidenced by their qualitative feedback. The I-CARE program shows potential in promoting mental health and emotional wellness among physicians in training.

**Tee et al 2020 (Research article)**	**Psychological impact of COVID-19 pandemic in the Philippines**	This study examined the prevalence of psychiatric symptoms and identified the factors contributing to psychological impact in the Philippines.	Focuses on depression, anxiety, stress, and the psychological impact of COVID-19 pandemic.	Online survey to collect primary data using scales, including Depression, Anxiety and Stress Scale - 21 Items (DASS-21) and Impact of Event Scale - Revised - 22 items (IES-R).	Adults-child population (N=1879) in general community settings, including Luzon, the Philippines.	28.8% of the respondents reported moderate-to-severe anxiety, 13.4% reported moderate-to-severe stress levels, 16.9% moderate-to-severe depression and 16.3% of respondents rated the psychological impact of the outbreak as moderate-to-severe (IES-R mean score 19.57 (SD=13.12), DASS-21 mean score 25.94 (SD=20.59)). Female gender, youth age of 12-21 years, single status, students, presence of specific physical symptoms (i.e., headache, cough, chills), recent imposed quarantine by a health authority, prolonged stay at home, poor self-reported health status, feeling of too much un-necessary worry has been made about COVID-19, concerns about family members getting sick, and feeling of being discriminated by other countries were associated with a greater psychological impact of the pandemic and higher levels of stress, anxiety and depression. Timely and accurate health information, having children older than 16 years old, perception of good health status and confidence in their own health care providers were associated with lesser psychological impact of the pandemic and lower levels of stress, anxiety and depression.

**Tee et al 2020 (Research article)**	**Psychological state and associated factors during the 2019 coronavirus disease (COVID-19) pandemic among filipinos with rheumatoid arthritis or systemic lupus erythematosus**	To present the psychological responses of Filipino Systemic Lupus Erythematosus and Rheumatoid Arthritis patients to the COVID-19 pandemic and shortage of hydroxychloroquine supply.	Investigated depression, anxiety, stress, psychological impact of COVID-19 pandemic. Found mean IES-R score higher among SLE (22.34, SD=14.39) than RA (18.85, SD=13.24) patients. Stress, anxiety and depression moderate to severe in 12.3%, 38.7%, and 27.7% of respondents. Mean stress subscale score 10.11 (SD=7.95), mean anxiety subscale score 6.79 (SD=6.57) and mean depression subscale score 9.03 (SD=8.77).	Online survey to collect primary data using the following: Depression, Anxiety and Stress Scale - 21 Items (DASS-21) and Impact of Event Scale - Revised - 22 items (IES-R), plus own survey.	Patient population: Systemic Lupus Erythematosus and Rheumatoid Arthritis (N=512) in urban, healthcare/ clinical setting in the Philippines.	In the third month of the pandemic in the Philippines, 20% of the respondents with lupus and rheumatoid arthritis experienced moderate to severe psychological impact. There was moderate to severe anxiety in 38.7% and moderate to severe depression in 27%. Risk factors for adverse mental health include the presence of comorbidity of hypertension and asthma; being a healthcare worker; and presence of specific symptoms of myalgia, cough, breathing difficulty, dizziness and sore throat, and protective factors include satisfaction with available health information and wearing of face masks.

**Tee et al 2021 (Research article)**	**Impact of the COVID-19 Pandemic on Physical and Mental Health in Lower and Upper Middle-Income Asian Countries: A Comparison Between the Philippines and China**	This study aimed to compare the levels of psychological impact and mental health between people from the Philippines (LMIC) andChina (UMIC) and correlate mental health parameters with variables relating to physical symptoms and knowledge about COVID-19.	Focuses on the psychological impact of the COVID-19 pandemic by looking at depression, anxiety, and stress.	Online survey to collect primary data using Depression, Anxiety and Stress Scale - 21 Items (DASS-21) and Impact of Event Scale - Revised - 22 items (IES-R).	Educator/learner population: network of university community (Ntotal=1710, NPhilippines=849) in educational setting: Huabei University and the University of the Philippines across 71 cities and 40 provinces in the Philippines (Luzon, Visayas, and Mindanao), and 159 cities and 27 provinces in China.	Filipino (LMIC) respondents reported significantly higher levels of depression (9.72), anxiety (7.30), and stress (10.60) than Chinese (UMIC) during the COVID-19 ($p<0.01$). IES-R was 20.67 for the Philippines and 30.76 for China. Filipino respondents were more likely to report physical symptoms resembling COVID-19 infection ($p<0.05$), recent use of but with lower confidence on medical services ($p<0.01$), recent direct and indirect contact with COVID ($p<0.01$), concerns about family members contracting COVID-19($p<0.001$), dissatisfaction with health information ($p<0.001$). Student status, low confidence in doctors, dissatisfaction with health information, long daily duration spent on health information, worries about family members contracting COVID-19, ostracization, and unnecessary worries about COVID-19 were associated with adverse mental health. The male gender and having a child were protective factors significantly associated with the lower score of IES-R ($p<0.05$) and depression ($p<0.001$), respectively. Single status was significantly associated with depression ($p<0.05$), and student status was associated with higher IES-R, stress and depression scores ($p<0.01$).

**Teng-Calleja et al 2020 (Research article)**	**Examining organizational response and employee coping behaviors amid the COVID-19 pandemic**	Using the crisis in context theory (CCT) as an ecological framework to understanding human behaviors, this study examined organizational responses and individual employee coping behaviors to mitigate the impact of the COVID-19 pandemic.	Researched coping behaviors to crisis on organizational and individual levels, to emphasize that responses to crises must be examined with the contextual or ecological perspective in mind. They discuss several interventions, such as flexible work arrangements, mental health programs, PPE, financial support, coping/stress management strategies.	Online survey to collect primary data, no scale provided.	Employee population: (N=216) in 25 different organizations across various settings in the Philippines: academe (12.04%), business process outsourcing (7.4%), manufacturing (6.48%) and government (6.48%), rest belonged to health, retail, utility, infrastructures, real estate, and non-profit organizations, among others.	This study identified organizational actions or responses to help employees adapt to the COVID-19 crisis: 1) flexible work arrangements, 2) mental health and well-being programs, 3) physical health and safety measures, 4) financial support, 5) provision of material resources, and 6) communication of short and long term plans. Findings also surfaced coping strategies at the individual employee level and how these relate to organizational initiatives: 1) task-focused coping, 2) stress management, 3) social coping, 4) cognitive strategies, 5) learning and development activities, 6) faith-oriented coping, and 7) maladaptive strategies. Findings reflect how policies and guidelines at the societal level shape organizational interventions and employee coping strategies.
**Toquero 2020 (Opinion editorial/commentary/viewpoint)**	**Inclusion of people with disabilities amid COVID-19: Laws, interventions, recommendations**	This article examines the laws of the Philippine Government on the provision for the inclusive special education and rights of the said learners, highlights the possible educational interventions to supplement their learning amid the pandemic and offers recommendations for the emergency preparedness legislative policies and services to be responsive to the educational, socio-emotional, and mental health needs of the students with disabilities amid the pandemic.	Brings up poor mental health, stress, loneliness, depression,and anxiety during COVID-19. Talks about laws that protect people with disabilities, interventions for people with special needs and disabilities (use of assistive technology, use of social media), proper emergency preparedness and mental health interventions from government for people with disabilities during pandemic times.	No collection method of primary or secondary data. Author cited "desktop analysis approach of the scientific body of literature focused on inclusive education to determine the existing provisions for people with disabilities."	People with disabilities (N=NA) in the Philippines.	This article gives social, academic, and policy implications. Socially, people with disabilities should be given protection and a platform where they can also experience that the government provides for their political and civil rights. Academically, teachers need to maximize the use of emergency remote teaching through assistive technologies so that there will be no interruption on the virtual-based education of the students with disabilities. Additional laws and policies need to be created to uphold the rights of people with disabilities since they too can experience numerous challenges on their education, mental health, and entire well-being due to the pandemic.

**Toquero 2021 (Opinion piece)**	**Provision of mental health services for people with disabilities in the Philippines amid Coronavirus outbreak**	To call on the need for the provision of mental health services for people with disabilities, and for the governments to have a rights-based dis-ability lens in their policy decision-making relative to emergency health response and recovery health plans amidst the corona virus outbreak in the Philippines.	Focused on stress, anxiety, depression, suicidal behavior, self-harm, and inclusive health responses.	NA	People with disabilities (N=NA) in general community settings across the Philippines.	Legislative policies need to protect the welfare of people with disabilities in the post-pandemic period, and there is a need for innovative research concerning the mental health needs and conditions of people with disabilities to examine and address the impact of COVID-19 to their mental health. Recommendations for mental health provision to fulfill the health-care needs of people with disabilities in the post-pandemic society: Inclusivity in emergency and recovery health response, ensuring rights of people with disabilities and more research, addressing health barriers through disability-inclusive recovery and legaslative policies, providing accessible health services and health support such as provision of digital health interventions through online health counseling and teleconferencing.
**Valenzuela et al 2022 (Literature review)**	**Impact of COVID-19 pandemic on sleep of undergraduate students: A systematic literature review**	To describe sleep problem rates and prevalence, sleep pattern disruption, sleep duration, sleep quality, insomnia symptoms, psychological and socio-economic factors affecting sleep of undergraduates in 22 countries.	About sleep problems, sleep pattern changes, sleep duration, sleep quality, insomnia rates, and psychosocial factors with sleep problems.	Document review to collect secondary data. No scales reported.	Educators/learners (N=NA) in educational settings in several countries, including the Philippines.	A considerable prevalence of undergraduates have encountered sleep problems during the pandemic. Moreover, sleep pattern disruption and increased sleep duration were consistently reported, while sleep quality and insomnia symptoms varied across included populations. In the Philippines, the mean sleep duration reportedly decreased during the pandemic from 7.5 to 6.7 h. The researchers also explicated the relationship between sleep and mental health during the pandemic.

**Wang 2021 (Research article)**	**A chain mediation model on COVID-19 symptoms and mental health outcomes in Americans, Asians and Europeans**	To test the model triggered by physical symptoms resembling COVID-19 infection, in which the need for health information and perceived impact of the pandemic mediated the path sequentially, leading to adverse mental health outcomes.	Addressed psychological impact of COVID-19, depression, anxiety and stress, and physical symptoms resembling COVID-19, and health information.	Online survey to collect primary data using the Depression, Anxiety and Stress Scale (DASS-21), the Impact of Event Scale-Revised (IES-R), and the National University of Singapore (NUS) COVID-19 ques-tionnaire.	Adult population (Ntotal=4612, NPhilippines=619) in various community settings across the Philippines, China, Iran, Pakistan, Poland, Spain, the United States of America, and Vietnam.	This study found that Poland and Pakistan were two countries with the highest mean scores in IES-R and DASS-21 anxiety, depression and stress scales. In contrast, Vietnam had the lowest mean scores in IES-R and DASS-21 anxiety, depression and stress scales, while Poland and the Philippines were the two countries with the highest levels of anxiety, depression and stress. The chain mediation model shows that the need for health information and the perceived impact of the pandemic exert sequential mediating effects on mental health outcomes in people who experience physical symptoms that resemble COVID-19 infection. Chain mediation model showed the need for health information, and the perceived impact of the pandemic were sequential mediators between physical symptoms resembling COVID-19 infection (predictor) and consequent mental health status (outcome). Excessive and contradictory health information might increase the perceived impact of the pandemic.

**Wang et al 2021 (Research article)**	**Gender differences in psychosocial status of adolescents during COVID-19: a six-country cross-sectional survey in Asia Pacific**	To describe the early experiences of adolescents with the COVID-19 pandemic: to examine if adolescents´ studying (i.e.,studying at home, receiving remote education, and receiving online courses) and leisure activities (i.e., playing physically, watching TV, playing video games, and sleeping in daytime), psychosocial status, and sources of COVID-19 information differed by gender in Bangladesh, India,Indonesia, Myanmar, the Philippines, and Vietnam in the Asia Pacific Region.	Addressed adolescent mental health, adolescents feeling isolated, stressed, and experiences during the stay-at-home orders, including 1) studying (i.e., all types of study activities), 2) remote education by school, 3) online courses, 4) playing-physically, 5) sleeping during daytime, 6) watching TV, and 7) playing games on TV, phones, and tablets. Also discussed negative psychosocial status.	Survey to inquire secondary data using no standardized scale.	Educator/learner population: adolescents, (Ntotal=12232, NPhilippines=421) in various community settings across the Philippines, Bangladesh, Cambodia, India, Indonesia, Laos, Mongolia, Myanmar, Nepal, Sri Lanka, Thailand, Timor-Leste, amd Vietnam.	Findings revealed that surveyed adolescents in six countries in the Asia Pacific region were experiencing a severe disruption of education and lack of access to distance learning. During the first year of the pandemic, the physical and psychosocial status of female youth were more negatively affected than male peers. Female adolescents in the Philippines and Vietnam had 2.22 times (95%CI: 1.14, 4.33,p=0.02) and 1.23 times (95%CI: 1.03, 1.47, p=0.02) higher odds about feeling unsafe or insecure than their male counter-parts.

**Wang et al 2021 (Research article)**	**The impact of COVID-19 pandemic on physical and mental health of Asians: A study of seven middle-income countries in Asia**	To compare the mental health status during the pandemic in the general population of seven middle income countries (MICs) in Asia (China, Iran, Malaysia, Pakistan, the Philippines, Thailand, and Vietnam).	Impact of COVID-19 event and Depression, Anxiety and Stress Scale<br><br>NA	Online survey to collect primary data using the revised Impact of Event Scale (IES-R), the 21-item Depression, Anxiety, and Stress Scale (DASS-21), and an online questionnaire about COVID-19 developed by National Univ of Singapore.	Adult population (Ntotal=4479, NPhilippines=851) in various community settings across the Philippines, China, Iran, Malaysia, Pakistan, Thailand and Vietnam.	This multinational study across 7 MICs in Asia showed that Thailand reported the highest mean IES-Rand DASS-21 anxiety, depression and stress scores. In contrast, Vietnamese reported the lowest mean scores in IES-R and DASS-21 anxiety, depression and stress scales. The Philippines had a high DASS-21 anxiety score (mean = 10.60, SD = 8.01), and a high depression score (mean = 9.72, SD = 8.99). The risk factors for adverse mental health include age <30 years, high education background, single and separated status, discrimination by other countries, contact with people with COVID-19 and worries about COVID-19. The protective factors for mental health include male gender, staying with children, staying with 6 or more people, employment, confidence in own´s doctors diagnosing COVID-19, high perceived likelihood of surviving COVID-19, spending less time on health information, hand hygiene practice and wearing a face mask.
