# Supplementary material for: The COVID-19 pandemic’s effects on mental and psychosocial health in the Philippines: A scoping review
Source: Glob Ment Health (Camb). 2024 Feb 8;11:e27. doi: 10.1017/gmh.2024.14 (PMC10988143; doi:10.1017/gmh.2024.14)
Supplement: Peer review history

# Editor report: Recommendation: The COVID-19 pandemic’s effects on mental and psychosocial health in the Philippines: A scoping review — R0/PR2

**DOI:** 10.1017/gmh.2024.14.pr2

**Affiliations:** School of Public Health, Universidad del Valle, Colombia

Dear Authors:

Please, follow the request of reviewers for a Major Revision.

Also, please review the manuscript with anti-plagiarism software for improving the writing.

Best regards.

# Editor report: Decision: The COVID-19 pandemic’s effects on mental and psychosocial health in the Philippines: A scoping review — R0/PR3

**DOI:** 10.1017/gmh.2024.14.pr3

**Affiliations:** Johns Hopkins University Bloomberg School of Public Health, United States

No accompanying comment.

# Editor report: Recommendation: The COVID-19 pandemic’s effects on mental and psychosocial health in the Philippines: A scoping review — R1/PR5

**DOI:** 10.1017/gmh.2024.14.pr5

**Affiliations:** School of Public Health, Universidad del Valle, Colombia

No accompanying comment.

# Editor report: Decision: The COVID-19 pandemic’s effects on mental and psychosocial health in the Philippines: A scoping review — R1/PR6

**DOI:** 10.1017/gmh.2024.14.pr6

**Affiliations:** Johns Hopkins University Bloomberg School of Public Health, United States

No accompanying comment.
